# Interferon regulatory factor 1 (IRF-1) promotes intestinal group 3 innate lymphoid responses during *Citrobacter rodentium* infection

**DOI:** 10.1038/s41467-022-33326-5

**Published:** 2022-09-29

**Authors:** Angelika Schmalzl, Tamara Leupold, Lucas Kreiss, Maximilian Waldner, Sebastian Schürmann, Markus F. Neurath, Christoph Becker, Stefan Wirtz

**Affiliations:** 1 Medizinische Klinik 1, Universitätsklinikum Erlangen, Friedrich-Alexander-Universität Erlangen-Nürnberg, Erlangen, Germany; 2 Institute of Medical Biotechnology, Friedrich-Alexander University Erlangen-Nürnberg, Erlangen, Germany; 3 Medical Immunology Campus Erlangen, FAU Erlangen-Nürnberg, Erlangen, Germany

**Keywords:** Intestinal diseases, Innate lymphoid cells, Mucosal immunology

## Abstract

Group 3 innate lymphoid cells (ILC3s) are crucial mediators of immunity and epithelial barrier function during immune responses against extracellular bacteria. Here, we identify Interferon regulatory factor 1 (IRF-1), a transcription factor previously associated with type 1 immunity, as an essential regulator of intestinal ILC3 accumulation and effector cytokine production. We demonstrate that IRF-1 is upregulated in the context of infection with the enteropathogen *Citrobacter rodentium* and that its presence is central for anatomical containment and prevention of pathogen dissemination. We furthermore show that IRF-1 is required in order for intestinal ILC3s to produce large amounts of the protective effector cytokine IL-22 early in the course of infection. On a molecular level, our data indicate that IRF-1 controls ILC3 numbers and their activation by direct transcriptional regulation of the IL-12Rβ1 chain, thereby allowing ILCs to physiologically respond to IL-23 stimulation.

## Abstract (web summary)

Innate lymphoid cells (ILC) are involved with different immune responses. Here the authors show that Interferon regulatory factor 1 (IRF1) is important for intestinal ILC3 accumulation during *Citrobacter rodentium* infection and promotes release of the protective cytokine IL-22 and response to IL-23.

## Introduction

Complex and tightly regulated immunological networks of both innate and adaptive immune cells provide intestinal homeostasis and, at the same time, confer effective protection against potentially invasive pathogenic threats. Type 3 innate lymphoid cells (ILC3s) are innate immune cells particularly enriched in gut associated lymphoid tissues (GALT) that are increasingly appreciated as gatekeepers of intestinal barrier integrity and immune homeostasis^1,2^. ILC3-derived cytokines directly interact with intestinal epithelial cells (IEC) and modulate other immune cell functions to orchestrate tissue reorganization. While they produce cytokines crucial for barrier protection, dysregulated activation of ILC3s can disrupt gut homeostasis and contribute to severe chronic pathologies such as inflammatory bowel disease (IBD) and colorectal cancer^3^. Although ILC3s are generally defined by the expression of the transcription factor RAR-related orphan nuclear receptor gamma t (RORγt), they are a rather heterogeneous group consisting of different subtypes which, in mice, can be broadly distinguished based on their expression of the C-C chemokine receptor type 6 (CCR6) and the natural cytotoxicity receptor (NCR) NKp46^4^. LTi-like ILC3s express CCR6 and are capable of secreting substantial amounts of IL-22 and IL-17. CCR6^-^NKp46^-^ ILC3s mainly produce IL-22 and were shown to differentiate in inflammatory settings through the Notch-dependent upregulation of Tbet into CCR6^-^NKp46^+^ ILC3s that mainly produce IFN-γ^5^. As a result of IL-12 and IL-18 stimulation, CCR6^-^NKp46^+^ ILC3s downregulate RORγt and develop into Tbet^+^ ex-RORγt ILC3s or ILC1-like cells^6^. It is believed that this phenotypical plasticity allows ILC3s to adaptively switch between inflammatory and homeostatic phenotypes in accordance with the current environmental conditions^7,8^.

The non-invasive attaching-effacing bacterial pathogen *Citrobacter rodentium* has been well appreciated as a model to study the processes that lead to the activation of innate and adaptive components of the intestinal immune system^9^ and serves as a model of human infections with enteropathogenic *E. coli* and enterohaemorrhagic *E. coli* (EPEC/EHEC) and IBD. During the early phase of infection, the cytokine IL-22 is essential to confer host protection and RORγt-expressing group 3 innate lymphoid cells (ILC3) have been identified as a critical cellular source of this cytokine^10^.

The transcription factor interferon regulatory factor 1 (IRF-1) is ubiquitously expressed at low basal levels, where it maintains constitutive expression of its target genes. During various infectious diseases, IRF-1 expression in cells is strongly induced by several factors including interferons or pathogen sensors such as TLRs, NLRs and RLRs^11,12^. In the human gut, IRF-1 upregulation has been associated with chronic intestinal inflammation^13,14^, while studies of *Irf1*^–/–^ mice in a mouse model of chemically induced colitis suggested protective roles during intestinal inflammation^15^. However, even though IRF-1 is well-known as an important contributor to immune defense mechanisms at multiple levels, the role of IRF-1 in innate immune responses at mucosal surfaces remains incompletely understood.

In this study, we characterize the function of IRF-1 during the early phase of intestinal inflammatory conditions. We demonstrate that IRF-1 is upregulated in the context of enteric infection with the gram negative model organism *C. rodentium* and is a central regulator of protective mucosal immunity in this model. We show that impaired IL-22 production by ILC3s in *Irf1*^*–/–*^ mice leads to insufficient intestinal immune protection and a lack of anti-bacterial defense during *C. rodentium* infection. On a molecular level, ILC intrinsic IRF-1 expression is essential for appropriate intestinal ILC3 activation by controlling their capacity to physiologically respond to IL-12 and IL-23 stimulation.

## Results

### IRF-1 expression is essential to mount efficient immune responses against *C. rodentium*

**Fig. 1:**
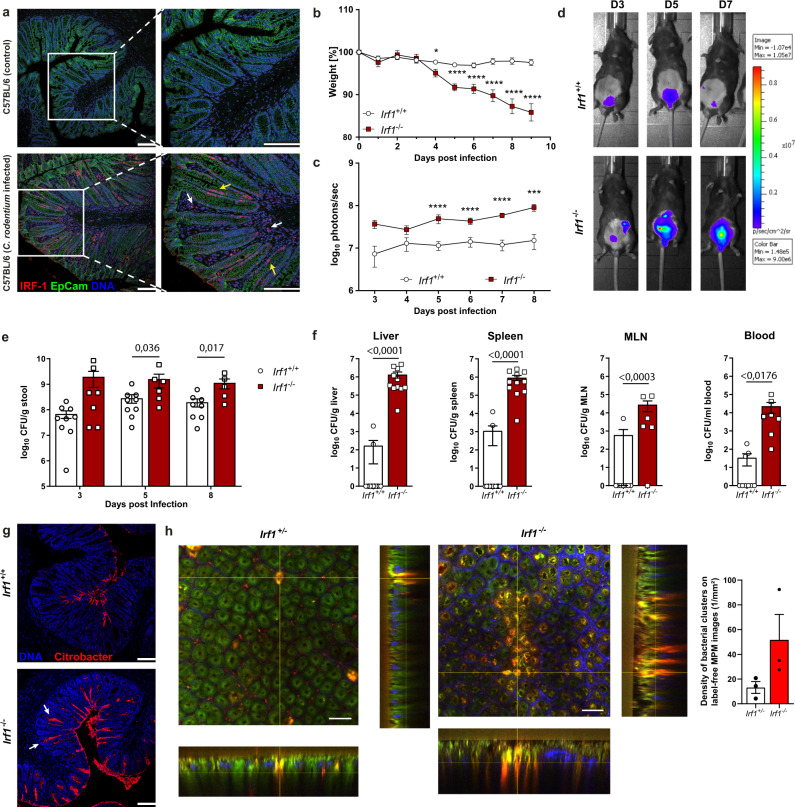
IRF-1 expression is essential to mount efficient C. rodentium directed immunity. **a** Colonic cross sections of uninfected or *C. rodentium* (9 dpi) infected C57BL/6 mice stained with anti-IRF-1 (red), anti-Ep-CAM (green), and DAPI (blue) and analyzed by confocal microscopy (epithelium: yellow arrow heads; lamina propria cells: white arrow heads). Scale bars represent 100 µm. **b**–**g** *Irf1*^+/+^ and *Irf1*^–/–^ mice were infected orally with 4 × 10^9^ CFU of *C. rodentium*. **b** Weights of infected mice were recorded daily and plotted as percentage of baseline (*Irf1*^+/+^: *n* = 19, *Irf1*^–/–^ *n* = 18). Day 4 pvalue: 0,0172; pvalues day5-day9: <0.0001. **c**, **d** Bacterial loads were measured via in vivo imaging (*n* = 15/group). Day 8 *p* value: 0.0008; *p* values day5–day7: <0.0001. **e** Fecal bacterial loads (CFU/g stool) on 3, 5 and 8 dpi. (*Irf1*^+/+^: *n* = 9, *Irf1*^–/–^ *n* = 7). **f** Dissemination of *C. rodentium* to livers (*Irf1*^+/+^: *n* = 13, *Irf1*^–/–^ *n* = 11), spleens (*Irf1*^+/+^: *n* = 13, *Irf1*^–/–^ *n* = 11), mLN (*Irf1*^+/+^: *n* = 8, *Irf1*^–/–^ *n* = 6) and blood (*Irf1*^+/+^: *n* = 8, *Irf1*^–/–^ *n* = 6) (9 dpi). **g** *C. rodentium* colonization of the colonic epithelial surface and penetration of the crypt bottom (white arrow heads) was visualized by staining cross sections by immunohistochemistry. Scale bar: 100 µm. **h** *Irf1*^+/+^ and *Irf1*^–/–^ mice (*n* = 3/group) were infected with a *C. rodentium* reporter-strain expressing M-Ruby-II. Tissue samples from distal colon (7 dpi) were analysed by label-free multiphoton microscopy (MPM; excitation at 810 nm) to define densities of bacterial clusters per MPM image (1/mm^2^). Scale bar: 50 µm. Data is expressed as mean ± SEM. Two-tailed Mann–Whitney U test was used for statistical comparison. In (**e**) and (**f**) exact *p* values are provided in the plots. *p ≤ 0.05; ***p ≤ 0.001; ****p ≤ 0.0001. Source data are provided as a Source data file.

Previous studies in human IBD and models of intestinal inflammation suggested important gut specific roles of IRF-1. Because immunohistochemical stainings demonstrated a profound increase in colonic IRF-1 protein during *C. rodentium* infection (Fig. 1a, Supplementary Fig. 1A), we took advantage of this widely utilized model to establish cell type specific functions of IRF-1 during infections with extracellular, noninvasive enteric pathogens. We therefore infected *Irf1*-deficient (*Irf1*^–/–^) mice with a luminescent *C. rodentium* strain and compared both the disease outcome and pathogen burden of these mice with *Irf1*^*+/+*^ controls. While control mice did not lose weight during the course of infection, *Irf1*^*–/–*^ *mice* substantially lost weight, starting from day four post infection (Fig. 1b). Notably, starting at 9 to 10 days post infection (dpi), severe disease in *Irf1*^*–/–*^ *mice* was evident by rapid weight loss and high mortality rates. To monitor bacterial loads during the course of disease in vivo, the pathogen-derived luminescence was measured daily using IVIS-based in vivo-imaging. Interestingly, detected luminescence intensities were significantly higher in *Irf1*^–/–^ mice at every time point of analysis (Fig. 1c, d) and correlated with increased fecal *C. rodentium* loads analyzed by plating of serially-diluted stool material on selective agar-plates (Fig. 1e). On day nine post infection, analysis of tissue homogenates from distal organs (liver, spleen and mesenteric lymph nodes (mLNs)) indicated only low systemic pathogen spread in controls, while *Irf1*^–/–^ mice displayed high systemic *C. rodentium* dissemination (Fig. 1f). *C. rodentium* typically attaches to superficial enterocytes that line the intestinal lumen, but does not cover IECs at the crypt base^16^. However, specific immunofluorescent staining demonstrated *C. rodentium* localization deep in colonic crypts of *Irf1*^–/–^ mice, whereas, control mice displayed pathogen signals only at the surface of the crypts as expected (Fig. 1g). To further characterize *C. rodentium*-induced mucosal lesions, we also used multiphoton microscopy with fresh colonic tissue^17^ to visualize infection-induced bacterial foci in infected mice. These experiments further confirmed an increased manifestation of mucosal *C. rodentium* clusters in *Irf1*^–/–^ mice and provided evidence that the absence of IRF-1 allows deep crypt penetration of the pathogen (Fig. 1h).

**Fig. 2:**
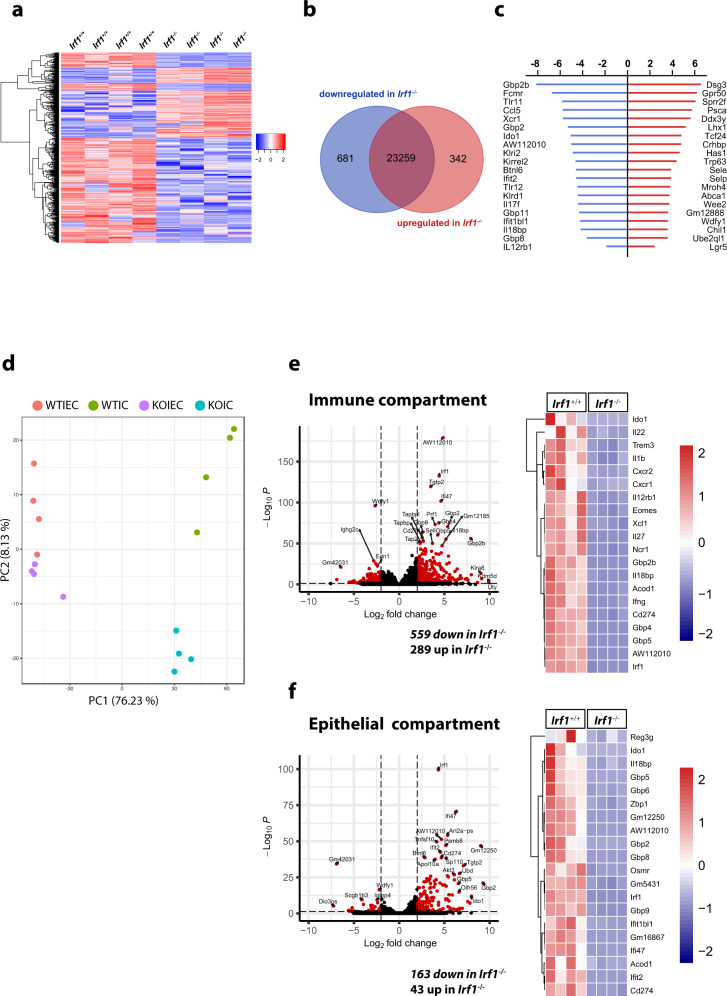
Analysis of Irf1-dependent transcriptional changes during C. rodentium infection. **a–c** Expression profiling by bulk RNAseq of distal whole colonic tissue of *C. rodentium* infected *Irf1*^*–/–*^ and *Irf1*^*+/+*^ mice (9 dpi). **d–f** Colonic tissue of *C. rodentium* infected mice (9 dpi) was removed and the epithelial and immune cell compartment was enriched as described in the methods section. Total RNA was isolated and used for expression profiling by bulk RNAseq analysis. **e**, **f** Genes showing log2 fold-changes in expression of >2 or –2 and false discovery rates (FDR) < 0.05 as determined by the Deseq2 algorithm were colored in red.

We next used RNAseq-based gene expression profiling to investigate the impact of IRF-1 inactivation on the global colonic transcriptome in whole colonic tissue of mice with *C. rodentium* infection. Thereby, unsupervised hierarchical clustering of normalized gene expression counts showed high similarities in transcriptional patterns within each experimental group, but substantial differences between the two experimental groups. (Fig. 2a). Accordingly, more than 1000 genes were differentially expressed between *Irf1*^–/–^ and *Irf1*^+/+^ mice (log2 fold change >2; *p* < 0.05). While the majority of these genes was downregulated in *Irf1*^–/–^ mice (681 out of 1023), 342 were upregulated (Fig. 2b). Consistent with a role of IRF-1 as negative regulator of cell cycle and cell proliferation, genes related to DNA-binding (*Trp63*) as well as several transcription factors (*Lhx1*, *Tcf24*) and the stem cell marker *Lgr5* were among the highly upregulated genes in *Irf1*^–/–^ mice. The group of the most downregulated genes included interferon-stimulated genes such as *Gbp2*, *Ido1*, *Ifit2*, *Gbp11*, *Ifit1bl1* or *Gbp8*. Interestingly, the expression of several genes involved in antibacterial defense mechanisms (e.g. *AW112010*^18^) and IL-12 responses such as *Tlr11* and *Tlr12*, which induce the production of IL-12 in DCs^19^ as well as the IL-12 receptor subunit *IL12rb1* were also downregulated in the absence of IRF-1 (Fig. 2c). Interestingly, bulk RNAseq analysis of either purified immune cells or epithelial cells separated from colons of the same *C. rodentium* infected mice identified *Irf1*-dependend gene expression programs in both intestinal cell compartments. Notably, the numbers of differentially expressed genes between *Irf1*^–/–^ and *Irf1*^+/+^ mice (log2 fold change >2; padj <0.05) was substantially higher in the immune cell compartment (Fig. 2d–f).

Given that IRF-1 is well known to molecularly act downstream of IFN-γ signaling, we also subjected mice lacking IFN-γ to *C. rodentium* infection. However, compared to control mice, we observed no marked weight loss and no increased gut pathogen loads in these animals (Supplementary Fig. 1 B,C). Although we observed a tendency towards an increased systemic spread, dissemination was much lower when compared to infected *Irf1*^–/–^ mice (Supplementary Fig. 1D) indicating that molecular mechanisms beyond type 2 interferon signaling contribute to the protective function of IRF-1 during *C. rodentium* infection.

Collectively, our data indicate that the transcription factor IRF-1 essentially contributes to effective *C. rodentium* eradication and prevents potentially fatal systemic spread in this model.

### Hematopoietic but not intestinal epithelial specific IRF-1 expression is required for effective control of *C. rodentium* infection

**Fig. 3:**
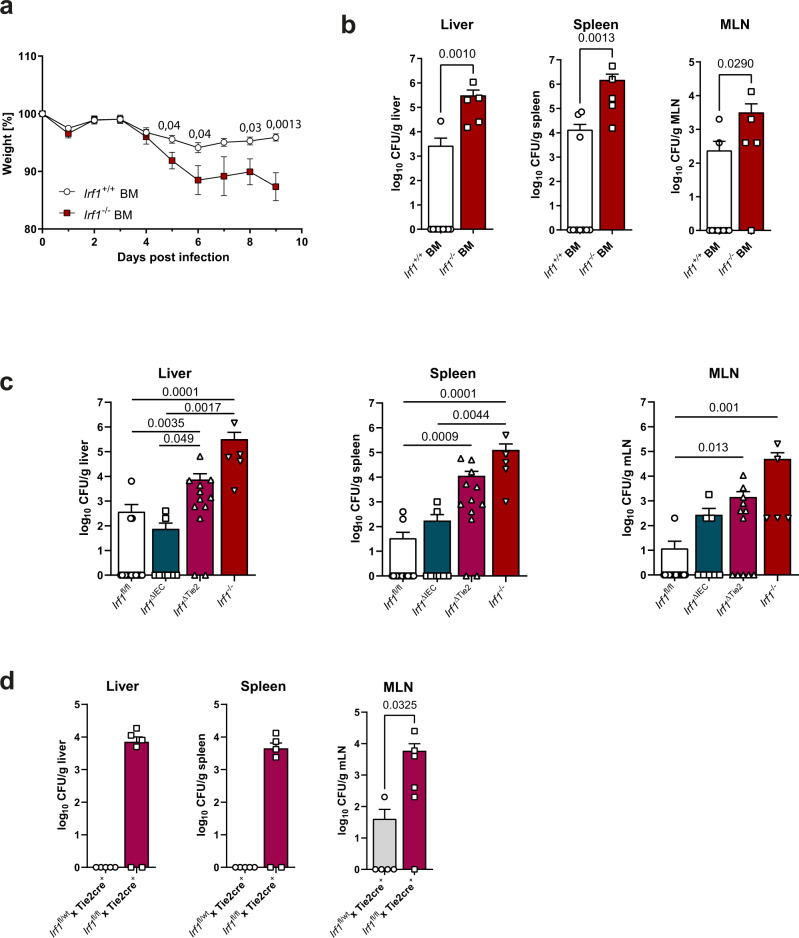
IRF-1 expression is required in hematopoietic cells during *C. rodentium* infection. **a**, **b** Chimeric mice were generated by reconstitution of irradiated C57BL/6 mice with *Irf1*^*+/+*^ or *Irf1*^*–/–*^ bone marrow. 8 weeks later, chimeras were infected with *C. rodentium* and analyzed 9 dpi. **a** Weights are shown as percentage of baseline (*Irf1*^+/+^ BM: *n* = 13, *Irf1*^–/–^ BM: *n* = 11). **b** *C. rodentium* dissemination to livers, spleens and mLNs. (*Irf1*^+/+^ BM: *n* = 10, *Irf1*^–/–^ BM: *n* = 5). **c** *Irf1*^–/–^, *Irf1*^ΔTie2^ and *Irf1*^ΔIEC^ mice were infected with *C. rodentium* and compared to *Irf1*^*fl/fl*^ mice. (*Irf1*^ΔTie2^: *n* = 12, *Irf1*^ΔIEC^: *n* = 8, *Irf1*^*fl/fl*^: n = 18, *Irf1*^–/–^: *n* = 5) **d** *Tie2*^Cre+^*Irf1*^fl/fl^ mice were infected and compared to *Tie2*^Cre+^*Irf1*^fl/wt^ control mice (*Tie2*^Cre+^*Irf1*^fl/fl^: *n* = 6, *Tie2*^Cre+^*Irf1*^fl/wt^: *n* = 5). Data is expressed as mean ± SEM. Exact *p* values defined by two-tailed Mann–Whitney U test (**a**, **b**, **d**) or by one-way ANOVA (Kruskal Wallis test) with Dunnett’s multiple comparisons test (**c**). Source data are provided as a Source data file.

Immunohistochemical stainings demonstrated that IRF-1 expression is strongly upregulated in both intestinal epithelial cells as well as lamina propria infiltrating immune cells in the context of *C. rodentium* infection (Fig. 1a). To discriminate between hematopoietic and non-hematopoietic contributions of IRF-1 expression during *C. rodentium* infection, we therefore next generated bone marrow chimeric mice by reconstituting lethally irradiated C57BL/6 mice with bone marrow from *Irf1*^*–/–*^ or *Irf1*^*+/+*^ mice and performed infection experiments eight weeks after reconstitution. Interestingly, C57BL/6 chimeras that received *Irf1*^*–/–*^ bone marrow suffered from marked wasting disease, while weight loss in the group of mice that were reconstituted with *Irf1*^*+/+*^ bone marrow was negligible (Fig. 3a). On day nine post infection, we observed systemic dissemination in all mice that had been reconstituted with *Irf1*^*–/–*^ bone marrow, while spread in the control group was significantly lower (Fig. 3b) suggesting that *Irf1* expression in the hematopoietic compartment is required to provide the host with the capacity to mount an efficient *C. rodentium* directed immune response. To further verify these results, we generated mice with conditional *Irf1*-deficiency in the hematopoietic and endothelial (*Irf1*^ΔTie2^) and in the intestinal epithelial (*Irf1*^ΔIEC^) compartments and compared the systemic spread of *C. rodentium* after infection in these mice. Our data clearly showed that *Irf1*^*ΔTie2*^ mice had higher systemic pathogen loads than *Irf1*^fl/fl^, *Tie2*^Cre^*Irf1*^+/–^ control and *Irf1*^ΔIEC^ mice (Fig. 3 c,d). Thus, IRF-1 expression in hematopoietic cells is required to prevent early bacterial overgrowth and systemic dissemination. Conversely, IRF1- expression in IECs seems to be less important in this setting, although expression of *Irf1* in IECs was highly upregulated in *C. rodentium*-infected C57BL/6 mice.

### IRF-1 deficiency impairs the effector functions of innate lymphoid cells during *C. rodentium* infection

**Fig. 4:**
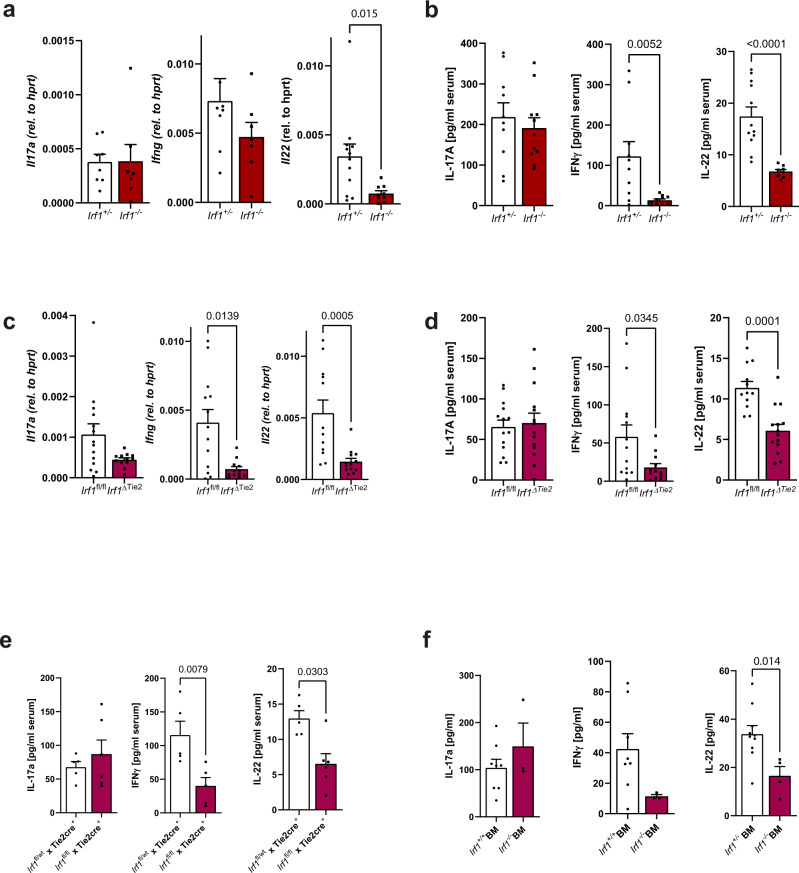
Irf1 deficiency impairs IL-22 and IFN-γ secretion during C. rodentium infection. **a**, **b** *Irf1*^–/–^ and control mice were infected with *C. rodentium*. **a** Total RNA of distal colonic tissue (8 dpi) was analyzed by specific qRT-PCR. (*Il17*,*Ifng*: *Irf1*^+/–^: *n* = 8, *Irf1*^–/–^: *n* = 7; *Il22*: *Irf1*^+/–^: *n* = 12, *Irf1*^-/–^: *n* = 9). **b** Serum-concentrations of IL-17A, IFN-γ and IL-22 were measured by specific ELISAs. (IL-17: *Irf1*^+/–^: *n* = 10, *Irf1*^-/–^: *n* = 11; IFN-γ: *Irf1*^+/–^: *n* = 10, *Irf1*^–/–^: *n* = 10; IL-22: *Irf1*^+/–^: *n* = 12, *Irf1*^–/–^: *n* = 8). **c**, **d**, **e** *Irf1*^ΔTie2^ and littermate control mice were infected with *C. rodentium*. **c** Total RNA of distal colonic tissue (8 dpi) was analyzed by specific qRT-PCR. (*Il17: Irf1*^fl/fl^: *n* = 14, *Irf1*^ΔTie2^: *n* = 12;,*Ifng*: *Irf1*^fl/fl^: *n* = 13, *Irf1*^ΔTie2^: *n* = 12; *Il22 Irf1*^fl/fl^: *n* = 12, *Irf1*^ΔTie2^: *n* = 13). **d** Serum-concentrations of IL-17A, IFN-γ and IL-22 were measured by specific ELISAs. (IL-17: *n* = 14/group, IFN-γ: *Irf1*^fl/fl^: *n* = 13, *Irf1*^ΔTie2^: *n* = 12, IL-22: *Irf1*^fl/fl^: *n* = 12, *Irf1*^ΔTie2^: *n* = 14). **e** Serum-concentrations of IL-17A, IFN-γ and IL-22 were measured by specific ELISAs. (IL-17, IL-22: *Irf1*^fl/+^ Tie2cre^+^: *n* = 5/group, *Irf1*^fl/fl^ Tie2cre^+^: *n* = 6, IFN-γ: *Irf1*^fl/+^ Tie2cre^+^: *n* = 5, *Irf1*^fl/fl^ Tie2cre^+^: *n* = 5). **f** Chimeric mice were generated by reconstitution of irradiated C57BL/6 mice with *Irf1*^*+/+*^ or *Irf1*^*–/–*^ bone marrow. 8 weeks later, chimeras were infected with *C. rodentium* and serum-concentrations of IL-17A, IFN-γ and IL-22 were measured by specific ELISAs (9 dpi). (IL-17, IFN-γ: *Irf1*^+/+^: *n* = 8, *Irf1*^–/–^: *n* = 3; IL-22: *Irf1*^+/+^: *n* = 10, *Irf1*^–/–^: *n* = 4). Data is expressed as mean ± SEM. Exact *p* values defined by two-tailed Mann–Whitney U test are provided in the plots. Source data are provided as a Source data file.

Characteristic cytokine secretion patterns of hematopoietic cells play essential roles during *C. rodentium* directed mucosal immunity^20^. To decipher the protective role of IRF-1 during the immune response against this pathogen in more detail, we next compared colonic cytokine expression profiles in infected *Irf1*^–/–^ and control mice at 8 dpi by specific qPCR analysis. While transcript numbers of IL-17A were comparable between control and *Irf1*-deficient mice, the transcripts of IFN-γ and particularly IL-22 were reduced in the absence of IRF-1 (Fig. 4a). Likewise, serum concentrations of IL-22 and IFN-γ, but not of IL-17A were significantly reduced in *Irf1*^–/–^ mice in comparison to controls (Fig. 4b). Notably, we observed a similar reduction of IFN-γ and IL-22 on mRNA and protein level in *C. rodentium* infected *Irf1*^ΔTie2^ mice compared to *Irf1*^fl/fl^ (Fig. 4c, d) or *Tie2*^Cre^*Irf1*^fl/wt^ mice (Fig. 4e) as well as in chimeric mice reconstituted with bone marrow cells of *Irf1*^–/–^ mice (Fig. 4f). By contrast, *Irf1*^ΔIEC^ mice exhibited similar IL-22 serum levels as *Irf1*^*fl/fl*^ controls.

**Fig. 5:**
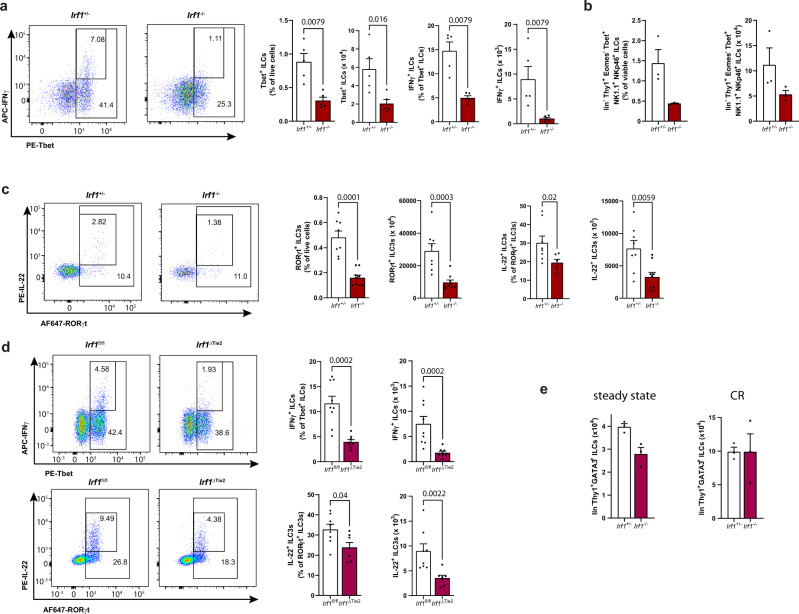
Irf1 expression regulates cytokine responses of intestinal innate lymphoid cells. **a–d** *Irf1*^–/–^ and *Irf1*^+/–^ control or *Irf1*^ΔTie2^ and *Irf1*^fl/fl^ control mice were infected with *C. rodentium*. LPMCs were isolated at 8 dpi and analyzed by flow cytometry. Graphs show relative abundances and frequencies of Tbet^+^ ILCs (lin^-^Thy1.2^+^Tbet^+^), IFN-γ producing Tbet^+^ ILCs (lin^-^Thy1.2^+^Tbet^+^IFN-γ^+^), ILC1s (lin^-^Thy1.2^+^Tbet^+^EOMES^-^NK1.1^+^Nkp46^+^) as well as relative abundances and frequencies of RORγt^+^ ILC3s (lin^-^Thy1.2^+^RORγt^+^) and ILC3s expressing IL-22 (lin^-^Thy1.2^+^RORγt^+^IL-22^+^). **a** *n* = 5/group; (**b**) *n* = 3/group; (**c**) *Irf1*^+/–^: *n* = 8, *Irf1*^+/–^: *n* = 9. **d** *Irf1*^fl/fl^: *n* = 9, *Irf1*^ΔTie2^: *n* = 8. **e** Comparison of intestinal ILC2 frequencies in *Irf1*^–/–^ and *Irf1*^+/–^ control mice in the steady state or in the context of *C. rodentium* infection (CR); *n* = 3/group. Data is expressed as mean ± SEM. Exact *p* values defined by two-tailed Mann–Whitney U test are provided in the plots. Source data are provided as a Source data file.

Both T cells and innate lymphoid cells (ILCs) have been shown to be important producers of IL-22 and IFN-γ during *C. rodentium* infection^21–23^. To characterize the differences in the expression patterns of these cytokines on a cellular level, we conducted flow cytometry analysis using colonic lamina propria mononuclear cells (LPMCs) of infected *Irf1*^*–/–*^ and *Irf1*^*+/–*^ control mice. In accordance with previous reports^24^, we observed reduced frequencies of T_H_1 cells (lin^+^Thy1^+^Tbet^+^ cells) in *Irf1*^*–/–*^ LPMCs. Interestingly, the frequencies of RORγt-expressing T cells (lin^+^Thy1^+^RORγt^+^ cells), which include T_H_17 and T_H_22 cells, were also reduced in these mice. However, intracellular cytokine stainings did not detect IRF-1-dependend differences in their capability to produce IL-22 and IFN-γ (Supplementary Fig. 1E, F, G). Within the innate lineage however, the frequencies of Tbet-expressing ILCs (lin^-^Thy1^+^Tbet^+^ cells) (Fig. 5a), ILC1s (lin^-^Thy1^+^Tbet^+^EOMES^-^NK1.1^+^NKp46^+^) (Fig. 5b) and RORγt-expressing ILC3s (lin^-^Thy1^+^RORγt^+^) producing IL-22 (Fig. 5c) were reduced in *Irf1*^*–/–*^ LPMCs compared to controls. In line with these findings, the abundances of IFN-γ- and IL-22-expressing ILCs were also significantly reduced in *Citrobacter* infected *Irf1*^*ΔTie2*^ mice (Fig. 5d). Because, the mouse intestinal lamina propria contains substantial numbers of ILC2s, we also analyzed the impact of *Irf1*-deficiency on this particular ILC subset. However, we observed no statistically significant differences between both genotypes in the steady state and in the context of *C. rodentium* infection (Fig. 5e).

Next, we adoptively transferred bone marrow of control or *Irf1*^–/–^ mice (both CD45.2^+^) to irradiated recipient mice harboring the congenic marker CD45.1 to allow discrimination of donor ILCs from radioresistant ILCs (Supplementary Fig. 2A). These experiments demonstrated that *C. rodentium* infected mice receiving *Irf1*^–/–^ bone marrow had significantly lower frequencies of intestinal IL-22 producing ILC3s, while Tbet^+^ ILCs were less affected. Supplementary Fig. 2B-D).

Although ILC3s in general require the transcription factors RORγt and AHR for development, phenotypically and functionally distinct subsets exist in the intestinal lamina propria^1,25^. In mice, the two best characterized groups can be distinguished by their expression of the chemokine receptor CCR6 and the natural cytotoxicity receptor NKp46^4,5^. Given the significant reduction of IL-22-producing ILC3s in *Irf1*^–/–^ mice, we therefore next analyzed, whether *Irf1*-deficiency differentially disturbs ILC3 subsets in the gut lamina propria. Already under steady state conditions, the relative abundance of CCR6^-^NKp46^+^ ILC3s was reduced in *Irf1*^–/–^ mice, while there were no significant changes in the frequencies of CCR6^+^NKp46^-^ ILC3s in LPMCs of *Irf1*^–/–^ mice (Supplementary Fig. 3A). Similarly, the numbers of CCR6^-^NKp46^+^ ILC3s were drastically reduced in the absence of IRF-1 in the context of *C. rodentium* infection (Supplementary Fig. 3B). The absolute numbers of CCR6^+^NKp46^-^ ILC3s were also reduced in *Irf1*^–/–^ mice indicating that IRF-1 transcription controls cellularity of both ILC3 subsets during infection (Supplementary Fig. 3B). Importantly, further flow cytometric analysis of LPMC after *C. rodentium* infection showed that all studied ILC3 subtypes of *Irf1*^*–/–*^ mice (CCR6^+^NKp46^-^, CCR6^-^NKp46^-^, CCR6^-^NKp46^+^) had a significantly diminished potential to produce IL-22 compared to control ILC3s (Supplementary Fig. 3C).

**Fig. 6:**
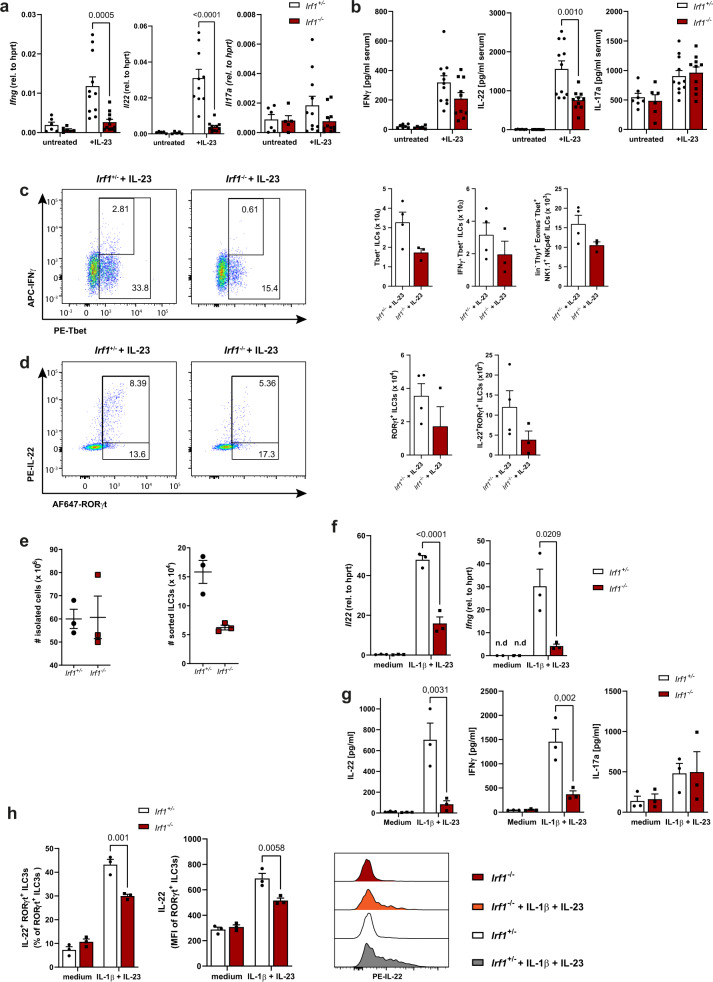
Irf1-/- ILCs fail to produce IL-22 and IFN-γ due to a cell intrinsic defect. **a–d** *Irf1*^*+/–*^ and *Irf1*^*–/–*^ mice were challenged with an IL-23-expression vector for three days. **a** The expression of *Ifng*, *Il22* and *Il17a* in gut tissue samples was quantified by qRT-PCR with total RNA (*Il17*: *Irf1*^+/–^ untreated *n* = 6, IL-23 *n* = 11; *Irf1*^–/–^ untreated *n* = 5, IL-23 *n* = 11; *Ifng*: *Irf1*^+/–^ untreated *n* = 6, IL-23 *n* = 11; *Irf1*^–/–^ untreated *n* = 6, IL-23 *n* = 11; *Il22*: *Irf1*^+/–^ untreated *n* = 5, IL-23 *n* = 10; *Irf1*^–/–^ untreated *n* = 5, IL-23 *n* = 11) and (**b**) serum-concentrations of IFN-γ, IL-22 and IL-17a were measured by specific ELISAs (IL-17: *Irf1*^+/–^ untreated *n* = 7, IL-23 *n* = 11; *Irf1*^–/–^ untreated *n* = 6, IL-23 *n* = 10; IFN-γ: *Irf1*^+/–^ untreated *n* = 7, IL-23 *n* = 11; *Irf1*^–/–^ untreated *n* = 6, IL-23 *n* = 10; IL-22: *Irf1*^+/–^ untreated *n* = 6, IL-23 *n* = 11; *Irf1*^–/–^ untreated *n* = 6, IL-23 *n* = 10). **c**, **d** Intestinal LPMCs were isolated and analyzed by flow cytometry. Graphs show frequencies of Tbet^+^ ILCs (lin^-^Thy1.2^+^Tbet^+^ cells), ILC1s (lin^-^Thy1.2^+^Tbet^+^EOMES^-^NK1.1^+^Nkp46^+^), ROR-γt^+^ ILC3s (lin^-^Thy1.2^+^RORγt^+^ cells) and of ILC3s expressing IL-22 (*Irf1*^+/–^: *n* = 4, *Irf1*^–/–^: *n* = 3). **e–h** *Irf1*^*+/–*^ and *Irf1*^*–/–*^ ILCs were sort-purified from LPMC before in vitro stimulation (*n* = 3/group). **e** Numbers of input cells and the yield of sort-purified ILCs (CD45^+^B220^-^CD3^-^CD5^-^CD11b^-^CD11c^-^KLRG1^-^CD127^+^Thy1.2^+^ cells) are shown. **f–h** ILCs were stimulated with IL-1β (20 ng/ml) and IL-23 (20 ng/ml) or left unstimulated (medium). **f** Total RNA of ILCs was analyzed by specific qRT-PCR. **g** After 72 h, supernatants were collected to measure concentrations of IL-17A, IL-22 and IFN-γ by ELISA. **h** IL-22 production of RORγt^+^ILCs was studied by flow cytometry. Data is expressed as mean ± SEM. Exact p-values defined by 2way ANOVA with Tukey´s multiple comparisons test are provided in the plots. n.d.: not detectable. Source data are provided as a Source data file.

Because expression of the cytokine IL-23 is essential for *C. rodentium*-directed mucosal immunity and intestinal ILC responses^26^, we next subjected control and *Irf1*^*–/–*^ mice to systemic IL-23 treatment and compared the expression of IFN-γ, IL-22 and IL-17A in this setting. Expectedly, IL-22 and IFN-γ were hardly detectable under steady state conditions. However, IL-23 treatment resulted in a strong increase of IL-22 and IFN-γ transcripts and protein in control mice, while this upregulation was blunted in *Irf1*^–/–^ mice (Fig. 6 a,b). Noteworthy, similar to the data in *C. rodentium* infected mice, the frequencies of Tbet^+^ IFN-γ-expressing and Rorγt^+^ IL-22-expressing ILCs were reduced in the absence of IRF-1 (Fig. 6 c,d). To more specifically analyze the cytokine responses of these cells in vitro, we flow-sorted lamina propria ILCs of *Citrobacter*-infected control and *Irf1*^–/–^ mice. Consistent with our previous flow cytometric data using LPMC, much less absolute numbers of ILCs were recovered from intestines of *Irf1*-deficient mice compared to controls **(**Fig. 6e). Although the expression of IL-22 and IFN-γ was very low in unstimulated ILCs, stimulation of control ILCs with a combination of IL-23 and IL-1β led to induction of IL-22 and to a lesser extend IFN-γ expression at both the RNA level (Fig. 6f, Supplementary Fig. 4A) and protein level (Fig. 6g, Supplementary Fig. 4B). By contrast, the induction of IL-22 and IFN-γ expression was strongly attenuated in *Irf1*^*–/–*^ ILCs upon stimulation of the same number of ILCs (Fig. 6f, g, h). Conversely, the stimulation-dependent secretion of IL-17A was not altered in *Irf1*^*+/–*^ and *Irf1*^*–/–*^ ILCs indicating that cell death is not a primary driver of the observed differences in cytokine expression patterns.

Collectively, these data indicate that *Irf1*-deficiency in the hematopoietic compartment is associated with a reduced capacity of intestinal lymphocytes to produce IL-22 and IFN-γ upon infection or IL-23 challenge. This phenotype is specifically linked to altered numbers and activation patterns within subsets of intestinal innate lymphoid cells in these mice.

### IRF-1 controls IL-23-dependent activation of intestinal ILC3s via IL-12Rβ1

**Fig. 7:**
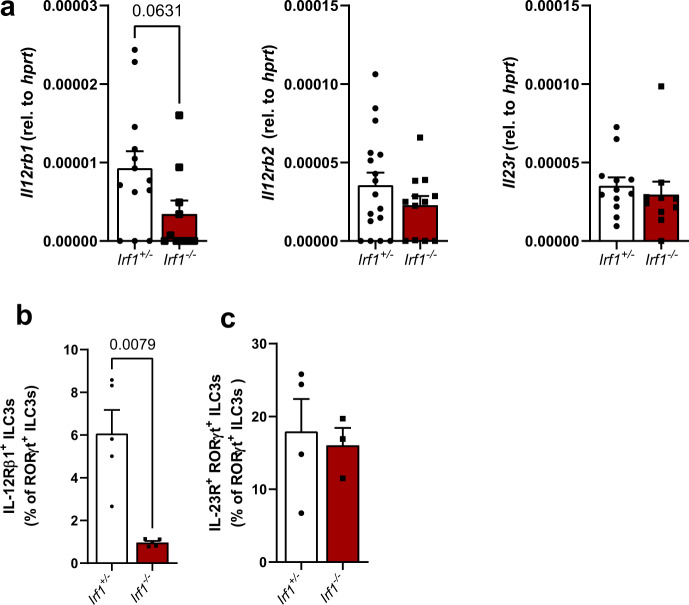
Reduced expression of IL12rb1 in Irf1-deficient intestinal ILC3s. *Irf1*^*+/*–^ and *Irf1*^*–/–*^ mice were infected with *C. rodentium* and were sacrificed eight days post infection. **a** Quantification of *Il12rb1* (*Irf1*^+/–^: *n* = 13, *Irf1*^–/–^: *n* = 10), *Il12rb2* (*Irf1*^+/–^: *n* = 17, *Irf1*^–/–^: *n* = 12) and *Il23r* (*Irf1*^+/–^: *n* = 12, *Irf1*^–/–^: *n* = 10) transcripts in total RNA of distal colon tissue samples by specific qRT-PCR. **b**, **c** Flow cytometric analysis of IL-12Rβ1 (*n* = 5/group) and IL-23R (*Irf1*^+/–^: *n* = 4, *Irf1*^–/–^: *n* = 3)-expressing ILC3s (lin^-^Thy1.2^+^RORγt^+^ cells). Data is expressed as mean ± SEM. Exact *p–* values defined by two-tailed Mann-Whitney U test are provided in the plots. Source data are provided as a Source data file.

In order to better define IRF-1-dependent transcriptional regulation of gut ILCs, we performed RNA-seq of FACS-sorted lamina propria ILC1/ILC3s of *C. rodentium*-infected *Irf1*^*–/–*^ and *Irf1*^*+/–*^ control mice. Principal component analysis showed that IRF-1-deficient ILCs clustered separately from their wild-type counterparts, demonstrating a unique role of IRF-1 in determining their transcriptome (Supplementary Fig. 5A). We identified more than 1000 genes (691 downregulated, 592 upregulated in *Irf1*^–/–^) as differentially expressed (FC > 2, adjp-value <0.05) between *Irf1*^–/–^ and control ILCs (Supplementary Fig. 5B). The list of highly downregulated genes includes several IFN-inducible genes such as *AW112010*, *Gbp2*, *Iigp1*, *Gm4951* and *F830016B08RiK*, but also genes previously implicated in ILC functions such as *Cysltr2 and Csf2*. Consistent with our flow cytometry data, we detected a downregulation of *IL22* transcripts in the absence of IRF-1. (Supplementary Fig. 5C). Furthermore, KEGG and GO enrichment analysis of differentially expressed genes (DEGs) between control and *Irf1*^-/-^ ILCs suggested that several DEGs are associated with important immune-related pathways including ‘Cytokine-cytokine receptor interaction’ (Supplementary Fig. 5D). The cell surface receptor IL-12Rβ1 forms with IL-12Rβ2 the receptor for the cytokine IL-12 or interacts with IL-23R to constitute a functional IL-23 receptor^27^. Interestingly, our RNA-Seq analysis of both sorted intestinal ILC3s and immune cells of *C. rodentium* infected mice clearly showed that the expression levels of *Il12rb1* were strongly reduced in *Irf1*^–/–^ mice. Because IL-12 and IL-23 receptor signaling have been shown to be central regulators of human ILC plasticity^28^ and IRF-1 transactivates the *Il12rb1* gene promoter in CD4^+^ T cells^29^, we compared the expression of this shared receptor chain in intestinal tissue of control and *Irf1*^–/–^ mice by qPCR. These data confirmed that *Il12rb1* expression was profoundly reduced in *Irf1*^–/–^ mice, while the transcripts of *Il12rb2* and *Il23r* were comparable between both groups of mice (Fig. 7a). Moreover, flow cytometric analysis clearly demonstrated that the presence of IRF-1 is required for IL-12rβ1 expression on intestinal ILC3s (Fig. 7b), while the IL-23R chain was similar in control and *Irf1*^–/–^ mice (Fig. 7c).

Collectively, these data indicate that IRF-1 is a critical regulator of intestinal ILC3 numbers and their functional potential by regulation of IL-12rβ1.

### IL-22 treatment protects *Irf1*^*–/–*^ mice from systemic *C. rodentium* dissemination

**Fig. 8:**
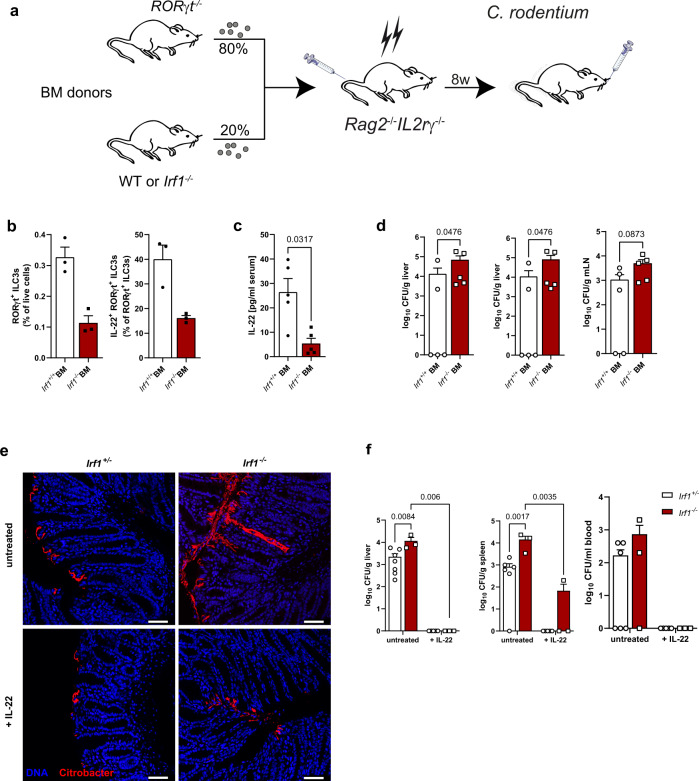
IL-22 treatment protects Irf1^–/–^ mice from systemic C. rodentium dissemination. **a** Mixed bone marrow (BM) chimeras with 80% RORγt^–/–^ (ILC3 deficient) and 20% control or *Irf1*^–/–^ bone marrow were generated. After 8 weeks, mice were infected with *C. rodentium* and analyzed at 8 dpi. **b** Flow cytometric analysis of ILC3s (lin^-^Thy1.2^+^RORγt^+^) and ILC3s expressing IL-22 (*n* = 3/group). **c** Serum IL-22 concentrations were measured by specific ELISA (*n* = 5/group). **d** Dissemination of *C. rodentium* was analyzed by determination of CFU/g tissue from livers, spleens and mLNs (*n* = 5/group). **e**, **f** Control and *Irf1*^–/–^ mice were injected with an *Il22* expression vector. After three days, mice were infected with *C. rodentium*. **e** *C. rodentium* colonization of the colonic epithelial surface was visualized by staining of colonic cross sections. Scale bars represent 50 µm. **f** Dissemination of *C. rodentium* was analyzed by determination of CFU/g tissue from livers (*Irf1*^+/–^: untreated *n* = 7, IL-22 *n* = 5; *Irf1*^–/–^: *n* = 3/group), spleens (*Irf1*^+/–^: untreated *n* = 6, IL-22 *n* = 5; *Irf1*^–/–^: *n* = 3/group) and CFU/ml blood (*Irf1*^+/–^: untreated *n* = 6, IL-22 *n* = 5; *Irf1*^–/–^: *n* = 3/group). Data is expressed as mean ± SEM. Exact *p* values defined by two-tailed Mann–Whitney U test (**b**–**d**) or 2way ANOVA with Tukey’s multiple comparisons test (**f**) are provided in the plots. Source data are provided as a Source data file.

In the first days after infection, ILC3s provide protection from *C. rodentium*-dependent disease^30^. Given the profound changes in the ILC3 compartment in *Irf1*^*–/–*^ mice, we next aimed to specifically address the contribution of ILC3 intrinsic *Irf1* expression to disease manifestation. We therefore generated mixed bone marrow chimeric mice lacking *Irf1* in ILC3s by adoptive transfer of bone marrow cells of ILC3-deficient *Rorgt*^–/–^ mice and *Irf1*^−/−^ mice mixed at a ratio of 80:20^31^ into irradiated, lymphopenic *Rag2*^*–/–*^*IL2rγ*^–/–^ mice (Fig. 8a). Control chimeras received 20% WT bone marrow in place. In these chimeras, all ILC3s were either WT or *Irf1*-deficient, while the majority of other hematopoietic and non- hematopoietic cells were *Irf1*-sufficient. At 8 dpi after *C. rodentium* treatment, IL-22^+^ ILC3s were reduced in mice receiving *Irf1*^–/–^ bone marrow (Fig. 8b) and accordingly, serum IL-22 concentrations were reduced (Fig. 8c). Moreover, these mice displayed higher systemic spread than controls receiving *Irf1*-proficient ILC3s (Fig. 8d) supporting the notion that *Irf1*-expression in ILC3s directly supports *C. rodentium* directed defense mechanisms. IL-22 production by ILCs has emerged as a central protective mechanism in the early phase of the *C. rodentium* infection model. Within ILCs, this function has been largely assigned to Lti-like CCR6^+^ ILC3s and their capacity to produce large amounts of IL-22 upon stimulation, while NKp46^+^ ILC3s producing IFN-γ were redundant even in the absence of T cells^32,33^. We therefore inferred that rather their strong ILC3-intrinsic defects to produce IL-22 than the lack of NKp46^+^ ILCs supports the observed fatal systemic spread in *Irf1*^–/–^ mice. To directly test this hypothesis, we increased the systemic abundance of IL-22^34^ in *Irf1*^*–/–*^ and control mice and compared the disease outcome with untreated mice. *C. rodentium* detection by immunohistochemical staining clearly demonstrated colonization of deep colonic crypt spaces in untreated *Irf1*^–/–^ mice compared to *Irf1*-proficient mice at day 7 post infection. Conversely, the pathogen was restricted to the top of the colonic crypts in *Irf1*^-/-^ and control mice subjected to IL-22 treatment (Fig. 8e). Moreover, IL-22 treatment almost completely prevented systemic *C. rodentium* spread even in the absence of IRF-1 as evidenced by analysis of tissue homogenates on selective agar plates (Fig. 8f) highlighting the essential impact of this cytokine for pathogen containment to the gut lumen.

## Discussion

In conclusion, this study provides further insights into the function of the transcription factor IRF-1 during enteric infections and potentially other gut diseases such as IBD. Even though IRF-1 is known to be an important driver of type 1 immunity against intracellular pathogens, we were able to show here that this transcription factor also plays indispensable tasks during type 3 immune responses following A/E enteropathogenic infection of the large intestine. In this context, we identified IRF-1 by analysis of conventional and novel conditional gene-deficient mice as central regulator of ILC1 and ILC3 cellularity and their capacity to produce prototypic cytokines. Thereby, IRF-1 expression was of particular importance for activation-dependent production of IL-22 by both CCR6^+^NKp46^-^ and CCR6^-^NKp46^+^ ILC3 subsets, which was related to prevention of *C. rodentium* dissemination. Mechanistically, our data indicate that IRF-1 signaling stimulates the expression of the shared IL-12Rβ1 chain in ILCs thereby providing ILC3s the capacity to respond to the cytokines IL-12 and IL-23, which are abundantly produced by myeloid cells in the course of enteric infections^35,36^. Previously, IRF-1 has been implicated in various effector functions of T cells including CD8^+^ T cells, Tr1 cells and Th1 cells^11^. Kano et al. found that differentiation of naive CD4^+^ T cells into IFN-γ producing Th1 cells was directly linked to IRF-1 binding to the *IL12Rb1* gene promoter, while Th17 cell differentiation was not affected in *Irf1*^–/–^ mice^29^. A potential mechanism to explain this differential requirement of IRF-1-dependent regulation of *IL12rb1* is that less IL-12Rβ1 is sufficient for IL-23-signaling, while higher levels are required for IL-12-induced Th1 differentiation. In line with this study, we found decreased frequencies of intestinal Th1 cells in infected *Irf1*^-/-^ mice, while the production of IL-22 and IL-17 by T cells was not profoundly affected. Conversely, our in vitro and in vivo data clearly indicate that the IL-23-dependent production of IL-22 strongly depends on the presence of IRF-1. IL-22 is a central mediator of protective immunity towards *C. rodentium* infection. While IL-22 release from both innate and adaptive cells is required for effective eradication of this pathogen^22^, ILC3s represent the main source of IL-22 in the early phase of infection^7^. The early severe phenotype of *Irf1*^–/–^ mice in this model together with the fact that ectopic IL-22 expression alone was sufficient to provide complete protection clearly suggest that IRF-1-dependent expression of IL-22 is critical for mucosal clearance of extracellular bacterial pathogens and reestablishment of homeostasis. However, although our study also identified IRF-1 as important fine tuner of the accumulation of intestinal NCR^+^ ILC subsets producing IFN-γ, these data support previous studies showing that NCR^+^ ILC3s are redundant in the context of *C. rodentium* infection^32,33^. Given their reported host protective functions e.g. during infections with *Salmonella enterica*^4^, *Toxoplasma gondii*^37^ or viral infections^38^, it therefore remains interesting to study the contribution of IRF-1 dependent IFN-γ producing ILCs in these models. It also still needs to be investigated, whether IRF-1 signaling supports pathogenic functions of IL-22 and IFN-γ producing ILC3s that have been reported in e.g. anti-CD40-induced innate colitis^39–41^. Future fate-mapping approaches and scRNA and/or scATAC-seq studies using wildtype and *Irf1*-deficient mice will help to provide a more comprehensive understanding of the function of IRF1 signaling for the transcriptional regulation of intestinal ILC plasticity and the identification of transcriptional targets in individual ILC populations including ILC1, exILC3 and different ILC3 subsets. Noteworthy, the human IRF-1 gene is located in the IBD5 susceptibility locus on chromosome 5, which is a risk haplotype associated with Crohn’s disease^42^. Thus, IRF-1 signaling may also be implicated in progression and aggravation of inflammatory bowel diseases by supporting the accumulation of dysregulated NCR^+^ ILCs, which has been reported in the inflamed gut mucosa in CD^43,44^.

## Methods

### Animals and housing conditions

*Irf1*^*–/–*^ mice^45^ were kindly provided by A. Kröger (University of Magdeburg). *Irf1*^*–/–*^ mice were bred with *Irf1*^*+/–*^ as littermate controls or were co-housed with *Irf1*^*+/+*^ mice for at least four weeks to ensure adaptation of the intestinal microbiome. No evident differences in α and β-diversity of the intestinal microbiota could be detected by 16S-based metagenomic sequencing (Supplementary Fig. 6A, B). *Ifng*^*-/-*^ mice were originally purchased from the Jackson Laboratory and were bred in-house. *Irf1*^*fl/fl*^ mice were generated in-house through crossing a mouse strain purchased from the European Mouse Mutant Archive (EMMA) of the European Conditional Mouse Mutagenesis Program (EUCOMM) with a general *FLP* deleter strain. *Irf1*^*fl/fl*^ mice were crossed to Tie2-cre mice or Villin-cre mice (Jackson Laboratory) to generate *Irf1*^*ΔTie2*^ and *Irf1*^Δ*IEC*^ mice respectively. All mice were bred on a C57BL/6 background and kept in individually ventilated cages. Mice of different experimental groups were age- and sex-matched. Sterile drinking water and food were provided *ad libitum*. Animal husbandry and experimental procedures were approved by the Government of Unterfranken (55.2.2-2532-2-712).

### *C. rodentium* infection model and quantification of the bacterial burden

To analyze the relevance of IRF-1 during infectious colitis, mice were infected with an erythromycin resistant and luminescent strain of *Citrobacter rodentium* (*C. rodentium;* strain ICC169) kindly provided by Christian Riedel^46^. Prior to infection, mice were fasted for 8 hours. *C. rodentium* was cultivated in sterile, erythromycin-supplemented (500 µg/ml) LB-medium at 37 °C with shaking and used for infection during the phase of exponential growth. For infection, bacteria were resuspended in sterile PBS and mice were inoculated with ~4 × 10^9^ CFU of *C. rodentium* in 200 µl PBS by oral gavage using a feeding needle. For analysis of bacterial burdens, *C. rodentium* luminescence was measured by in vivo-imaging using an IVIS Spectrum CT system (PerkinElmer, Waltham, Massachusetts). Therefore, the abdomen of the infected mice was depilated and they were anesthetized with 1,5-2% isoflurane. Quantification of luminescence was performed using the IVIS-associated software *Living Image 4.0*. For determination of *C. rodentium* in feces, fresh stool samples were collected and weighed. To quantify *C. rodentium* CFUs in liver, spleen or mLN, fresh tissue samples were weighed, covered with LB medium (1 ml LB medium per 0.1 g tissue) and homogenized in a mixer mill (MM 400, Retsch, Germany) at a frequency of 25 Hz for 2 min. Serial dilutions of dissolved stool pellets, blood or tissue homogenates were plated on erythromycin-supplemented LB-Agar plates. After an incubation time of 20 h at 37 °C, *C. rodentium* colonies were counted.

### Bone marrow chimeric mice

For the generation of bone marrow chimeric mice, lethally irradiated (10 Gray) C57BL/6 mice or congenic B6.SJL mice were reconstituted with 1 × 10^7^ femoral bone marrow cells of either *Irf1*^*+/+*^ mice or *Irf1*^*-/-*^ mice via i.v. injection. In some experiments, *Rag2*^–/–^*γc*^–/–^ were irradiated with a dose of 5 Gray and i.v. transferred with mixed donor bone marrow containing 80% *Rorgt*^–/–^ and 20% wildtype or *Irf1*^–/–^ bone marrow cells. Reconstituted mice were treated with antibiotics (Borgal, Virbac, France) for 2–3 weeks to prevent infections in the recovery phase. Eight weeks after hematopoietic reconstitution, bone marrow chimeric mice were analyzed in the steady state or infected with *C. rodentium*.

### Isolation of lymphocytes from the lamina propria and from mesenteric lymph nodes

Single cell suspensions from mesenteric lymph nodes were prepared through digestion with Collagenase B (0.25 mg/ml; Roche) and DNase I (0.05 mg/ml Roche) using a gentleMACS Octo Dissociator (program: 37c_m_SDK_1; Miltenyi Biotec) according to the manufacturer’s recommendations. For the isolation of lamina propria mononuclear cells (LPMCs) intestinal tissue was removed and cleaned from residual fat. Luminal contents were flushed out and the intestinal tissue was cut longitudinally and then laterally into pieces of 5 mm length. LPMCs were isolated with the lamina propria dissociation kit mouse from Miltenyi Biotec according to the manufacturer’s instructions under use of a gentleMACS Octo Dissociator (Miltenyi Biotec) running the program m_intestine_01. After the isolation process, the cell suspension was proceeded to Percoll gradient centrifugation (40%/80%) for purification. Epithelial cells for bulk RNAseq analysis were isolated by incubating longitudinally opened and cleaned intestinal tissue in PBS with 1 mM DTT for 10 min and subsequently in 20 ml of prewarmed HBSS with 1.5 mM EDTA for 15 min. Next, the tissue was vortexed for 1–2 min at maximum speed and intraepithelial lymphocytes were removed using Percoll gradient centrifugation (40%/80%). The pellet containing intestinal epithelial cells was centrifuged for 10 min at 400 g and the pellet was immediately subjected to RNA extraction.

### Flow cytometry

Prior to staining with antibodies against specific intra- or extracellular markers, freshly isolated LPMCs were incubated with anti-CD16/CD32 antibodies (anti-Fc-receptor; clone 93; eBioscience) for 10 min at 4 °C to block unspecific binding. To distinguish between cells of the innate or the adaptive compartment, LPMCs were incubated with a cocktail of biotinylated lineage antibodies including anti-B220 (RA3-6B2; eBioscience, 1:50), anti-CD3 (REA641, 1:50), anti-CD5 (1:50), anti-GR1 (RB6-8C5; eBioscience, 1:50), anti-SiglecF (REA798, 1:50) and anti-Ter119 (Ter-119, 1:50) for 10 min at 4 °C. After washing, streptavidin conjugated Brilliant Violet 421 (BioLegend, 1:666) or VioBright FITC (Miltenyi Biotec, 1:100) was applied for 30 min at 4 °C in a secondary staining combined with a selection of the following antibodies used for surface staining: anti-Thy1.2 (CD90.2 in PerCP-Vio700, 1:20), anti-CCR6 (CD196 in PE, 1:20), anti-NKp46 (REA815 in FITC, 1:50), anti-Eomes (REA116 in PE, Miltenyi, 1:10), anti-NK1.1 (PK136 in BV 421, 1:20) anti-IL23R Ab (FAB16861R in AF 647, R&D Systems, 1:66) and anti-IL-12Rβ1 (CD212 in PE; BD Pharmingen, 1:20). Subsequently, cells were fixed and permeabilized using the FoxP3 Transcription Factor Staining Buffer Set (Invitrogen) according to the manufacturer’s instructions followed by intracellular transcription factor staining with fluorochrome-coupled anti-RORγt (Q31-378 in AlexaFluor 647 or BV421; BD Pharmingen, 1:80), anti Gata3 (REA174, Miltenyi Biotec, 1:10) and anti-Tbet (eBio4B10 in PE; eBioscience, 1:40) antibodies (30 min at 4 °C). For intracellular cytokine measurements, anti-IL-22 (Poly5164 in PE; BioLegend, 1:20), anti-IL-17A (TC11-18H10.1 in PE/Cyanine7; BioLegend, 1:20) and anti-IFN-γ (XMG1.2 APC; eBioscience, 1:158) antibodies were applied in combination with the transcription factor staining. In this case, cells were stimulated with Cell Stimulation Cocktail plus protein transport inhibitors (eBioscience) according to manufacturer’s instructions for 4 h prior to antibody staining. Antibodies for flow cytometry were purchased from Miltenyi Biotech, unless specified otherwise. Samples were measured on an LSRFortessa cell analyzer (BD Biosciences, FacsDiva Software version 6.1.3) and evaluated with FlowJo 10 (FlowJo LLC). The gating strategy for characterization of the various immune cell populations is outlined in Supplementary Fig. 7.

### ILC sort

To purify intestinal ILCs for analysis in cell culture or for RNASeq analysis, we performed fluorescence activated cell sorting using a MoFlo Astrios EQ device (Beckman Coulter) within the Core Unit Cell Sorting Erlangen. ILCs were purified using the following surface marker panel: CD45^+^ (REA737 in VioBlue, 1:50), B220^-^ (REA755 in FITC, 1;50), CD3^-^ (REA641 in FITC, 1:50), CD5^-^ (in FITC, 1:50), CD11b^-^ (REA592 in APC-Vio770, 1:50), CD11c^-^ (REA754 in APC-Vio770, 1:50), KLRG1^-^ (2F1 in APC; eBioscience, 1:158), CD127^+^ (IL-7Rα; A7R34 in PE; BioLegend, 1:20), Thy1.2^+^ (in PerCP-Vio700, 1:20). Unless specified otherwise, all antibodies used for cell sorting were purchased from Miltenyi Biotec.

### In vitro ILC stimulation

To assay the cytokine responses of ILC3s ex vivo, ILCs were sort purified. Therefore, freshly isolated single cell suspensions from ileum, colon and mLNs of two mice of either *Irf1*^*+/–*^ or *Irf1*^*–/–*^ genotype were pooled and stained prior to cell sorting. For in vitro stimulation, 2 × 10^4^ sorted CD45^+^ B220^-^ CD3^-^ CD5^-^ CD11b^-^ CD11c^-^ KLRG1^-^ CD127^+^ Thy1.2^+^ cells were plated in 200 µl DMEM GlutaMAX medium (Gibco) supplemented with 10% FBS (Gibco), 1× MEM nonessential amino acids (Gibco), 1 mM sodium pyruvate (Gibco), 20 mM Hepes (Carl Roth), 50 µM 2-mercaptoethanol (Sigma-Aldrich) and 1 % penicillin-streptomycin (Sigma-Aldrich) in the presence of 20 ng/ml recombinant mouse IL-1β (ImmunoTools) and 20 ng/ml recombinant mouse IL-23 (Miltenyi Biotec). After 24 h or 72 h, culture supernatants were harvested for determination of cytokine concentrations by ELISA and residual ILCs were lysed for isolation of RNA.

### Gene expression analysis

Tissue pieces from distal colon or ileum were snap-frozen in liquid nitrogen and stored at −80 °C for RNA isolation. RNA was isolated from tissue using the NucleoSpin RNA Plus kit (#740984, Macherey-Nagel) according to the manufacturer’s recommendations. To analyze gene expression of sort purified ILC3s, we used the peqGOLD Micro Spin Total RNA Kit (#13-6831, VWR) following the manufacturer’s instructions. cDNA was synthesized with the Script RT-PCR kit (#PCR-511L, Jena Bioscience, Germany). Gene expression was analyzed via quantitative real-time PCR (qRT-PCR) using pre-designed QuantiTect Primer assays (Qiagen) in a CFX Connect System (Bio-Rad). ΔCT values were calculated to illustrate the expression of the indicated genes relative to the housekeeping gene *hypoxanthine phosphoribosyltransferase 1* (*hprt*).

### Bulk RNAseq

To analyze total transcriptome profiles of whole colon tissue (Fig. 2a–c) after *C. rodentium* infection, we isolated total RNA from the distal colon of *Irf1*^*+/+*^ and *Irf1*^*-/-*^ mice 9 d after infection. For applying the NucleoSpin RNA Plus kit (Macherey-Nagel). A total amount of 2 μg RNA per sample was used for the generation of sequencing libraries using the NEBNext Ultra RNA Library Prep Kit for Illumina (NEB) following manufacturer’s instructions. Library preparations were sequenced on an Illumina platform and paired-end reads were generated. Paired-end clean reads were mapped to the reference genome (mm10) using HISAT2 (v.2.0.5) software with default parameters^47^. FeatureCounts (v.1.6.4) was used to count the read numbers mapped to each gene^48^. Differential expression analysis between two conditions with 4 biological replicates per condition was performed using DESeq2 (v.1.22.2)^49^. The resulting p values were adjusted using the Benjamini and Hochberg’s approach for controlling the False Discovery Rate (FDR). Genes with an adjusted p value < 0.05 found by DESeq2 were assigned as differentially expressed.

### Cytokine measurements by Enzyme-linked immunosorbent assay (ELISA)

To quantify the concentrations of IL-22, IFN-γ and IL-17A in sera and cell supernatants, Ready-SET-Go ELISA Sets from eBioscience were used according to the manufacturer’s instructions.

### 16 S Next generation sequencing of fecal microbiota

Fecal samples were collected and immediately stored at −80 °C. Genomic bacterial DNA was isolated with the PSP Spin Stool DNA Kit (Stratec molecular, Germany) according to manufacturer’s recommendations. 10 ng of DNA was used to amplify the V3-V4 region of bacterial 16sRNA genes using the NEBNext Q5 Hot Start HiFi PCR Master Mix (NEB). Amplified PCR products were isolated with AMPure XP Beads (Beckmann Coulter Genomics), purified, pooled and subjected to 2 × 300 bp paired-end sequencing on an Illumina MiSeq platform^50^.

### Immunohistochemistry

For immunohistochemical (IHC) stainings, distal colonic tissues from mice infected with *C. rodentium* were fixed in buffered formalin (Roti-Histofix; Carl Roth) at 4 °C for 24 h, dehydrated, and embedded in liquid paraffin. 3-µm sections were cut using a microtome (Leica) and processed for IHC applying the Tyramide Signal Amplification (TSA) Cy3 system (Perkin Elmer) according to the manufacturer’s protocol. To analyze the expression level of IRF-1, a primary antibody from Cell Signaling (D5E4) was used (1:50 dilution). To visualize the colonization of the mucosal surface with *C. rodentium*, a primary antibody from Abcam (ab37056, 1:1000) was applied. Both primary antibodies were used in combination with a goat-anti-rabbit biotinylated secondary antibody (Jackson Immuno Research). Epithelial cells were stained with Alexa Fluor 488 anti-mouse CD326 (Ep-CAM; G8.8; BioLegend, 1:100). Nuclei were counterstained with DAPI (Invitrogen). Pictures were acquired on a Leica TCS SP5 II confocal microscope using Leica LAS AF version 2.7.3.9723 software.

### Multiphoton microscopy

Infected animals were sacrificed and fresh tissue samples from distal colons were kept in ice-cold PBS, and multiphoton microscopy (MPM) was performed on the same day. In total, 42 3D image stacks (nKO = 18, nhet = 24) were recorded and analysed from six animals (*n* = 3/group). An upright Multiphoton microscope (TriMScope II, LaVision BioTec, Bielefeld, Germany) was used in combination with a water immersion objective (HC Fluortar L 16x/0.6 W VISIR, Leica microsystems, Wetzlar, Germany) and a femtosecond-pulsed Ti-Sapphire laser (Chameleon Vision II, Coherent, Santa Clara, CA, USA), at a wavelength of 810 nm. Exponential z adaptation of the laser power was used to compensate for attenuation at greater tissue depths. As previously described, the optical filters were chosen to target second harmonic generation (SGH) from collagen-I (ET405/20, Chroma, Vermont, USA), and natural autofluorescence from NADH in mucosal epithelial cells (450/70 BrightLine HC, Semrock Inc., Rochester, NY, USA)^17^. In addition to that, a third channel was used to target fluorescence from mRuby-expressed by the reporter bacteria. MPM stacks were recorded at an axial spacing of 2 µm. The lateral image size was 682 × 682 µm², separated into 512 × 512 or 1024 × 1024 pixels. The image contrast was adjusted manually using Fiji/ImageJ 1.5 upon visual inspection. No further image processing has been used. The number of bacterial clusters was counted manually in each image and divided by the area of the field of view in order to obtain the cluster density. The average cluster density per sample was calculated from all six images of the respective sample.

### Statistics

Experimental results were plotted and analyzed for statistical significance with GraphPad Prism 8.3 (GraphPad Software Inc.), Excel 2016 and R 4.2.0. Data are shown as mean ± SEM. For comparison of two independent experimental groups, a two-tailed Mann–Whitney U test was used. If more than two groups were compared, one-way ANOVA (Kruskal Wallis Test) with Dunnett´s multiple comparisons test or 2way ANOVA with Tukey´s multiple comparisons test was performed. Differences of *p* ≤ 0.05 were considered as statistically significant indicated by asterisks (*p ≤ 0.05; **p ≤ 0.01; *** *p* ≤ 0.001; ****p ≤ 0.0001).

### Reporting summary

Further information on research design is available in the Nature Research Reporting Summary linked to this article.

## Supplementary information

Supplementary Information

Reporting Summary

## Data Availability

The RNA sequencing of this study have been deposited in the Sequence Read Archive (SRA) database of the NCBI under the Bioproject accession number: PRJNA705051. All other data generated during the current study are available from the corresponding author on reasonable request. Source data are provided with this paper.

## Source data

Source Data
