# Supplementary material for: Interferon regulatory factor 1 (IRF-1) promotes intestinal group 3 innate lymphoid responses during Citrobacter rodentium infection
Source: Nat Commun. 2022 Sep 29;13:5730. doi: 10.1038/s41467-022-33326-5 (PMC9522774; doi:10.1038/s41467-022-33326-5)
Supplement: Supplementary file 1 — Supplementary Information [file 41467_2022_33326_MOESM1_ESM.pdf]

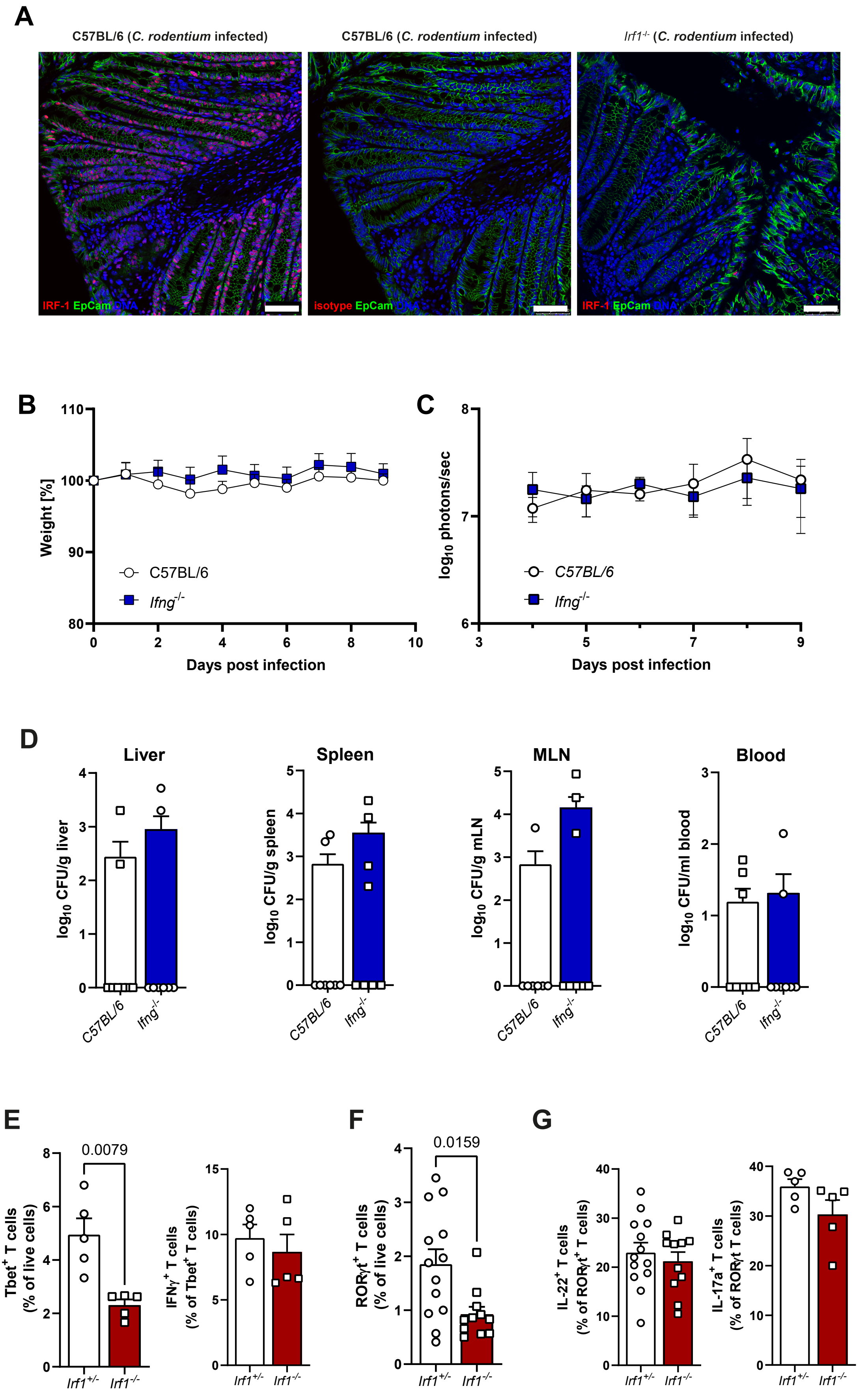

A
C57BL/6 (C. rodentium infected)
C57BL/6 (C. rodentium infected)
Irf1-/- (C. rodentium infected)
IRF-1 EpCam DNA
isotype EpCam DNA
IRF-1 EpCam DNA
B
Weight [%]
C57BL/6
Ifng-/-
Days post infection
C
log10 photons/sec
C57BL/6
Ifng-/-
Days post infection
D
Liver
log10 CFU/g liver
Spleen
log10 CFU/g spleen
MLN
log10 CFU/g mLN
Blood
log10 CFU/ml blood
C57BL/6
Ifng-/-
E
0.0079
Tbet+ T cells (% of live cells)
IFNγ+ T cells (% of Tbet+ T cells)
Irf1+/-
Irf1-/-
F
0.0159
RORγt+ T cells (% of live cells)
G
IL-22+ T cells (% of RORγt+ T cells)
IL-17a+ T cells (% of RORγt T cells)

Supplementary Figure 1

(A) Colonic cross sections of *C. rodentium* (9 dpi) infected C57BL/6 or *Irf1*$^{-/-}$ mice stained with anti-IRF-1 (red) or isotype control (middle panel), anti-Ep-CAM (green), and DAPI (blue) and analyzed by confocal microscopy. Scale bars represent 50 µm. **(B-D)** *Ifng*$^{-/-}$ mice were infected with *C. rodentium* and compared to C57BL/6 mice. (B,C) Weight curves are shown as percentage of baseline (n=8/group) and the bacterial load (n=4/group) was measured by *in vivo*-imaging using an IVIS Lumina II system. (D) Dissemination of *C. rodentium* was analyzed by determination of CFU/g tissue from livers, spleens and mLNs as well as of CFU/ml blood 9 dpi (n=8/group). **(E-G)** *Irf1*$^{+/-}$ and *Irf1*$^{-/-}$ mice were infected with *C. rodentium* and at 8 dpi, LPMCs were isolated from the colon and analyzed by flow cytometry. (E) Graphs show Tbet^{+} T cells (lin^{+} Thy1.2^{+} Tbet^{+} cells) and relative abundances of IFN-γ^{+} Tbet^{+} T cells (n=5/group). (F) Frequencies of RORγt^{+} T cells (lin^{+} Thy1.2^{+} RORγt^{+} cells; *Irf*1$^{+/-}$: n=13, *Irf*1$^{-/-}$: n=11) and (G) relative abundances of T cells expressing IL-22 (*Irf*1$^{+/-}$: n=13, *Irf*1$^{-/-}$: n=11) and IL-17A (n=5/group. Data is expressed as mean ± SEM. Exact p values ≤ 0.05 as defined by two-tailed Mann-Whitney U test are provided in the figure plots. Source data are provided as a Source data file.

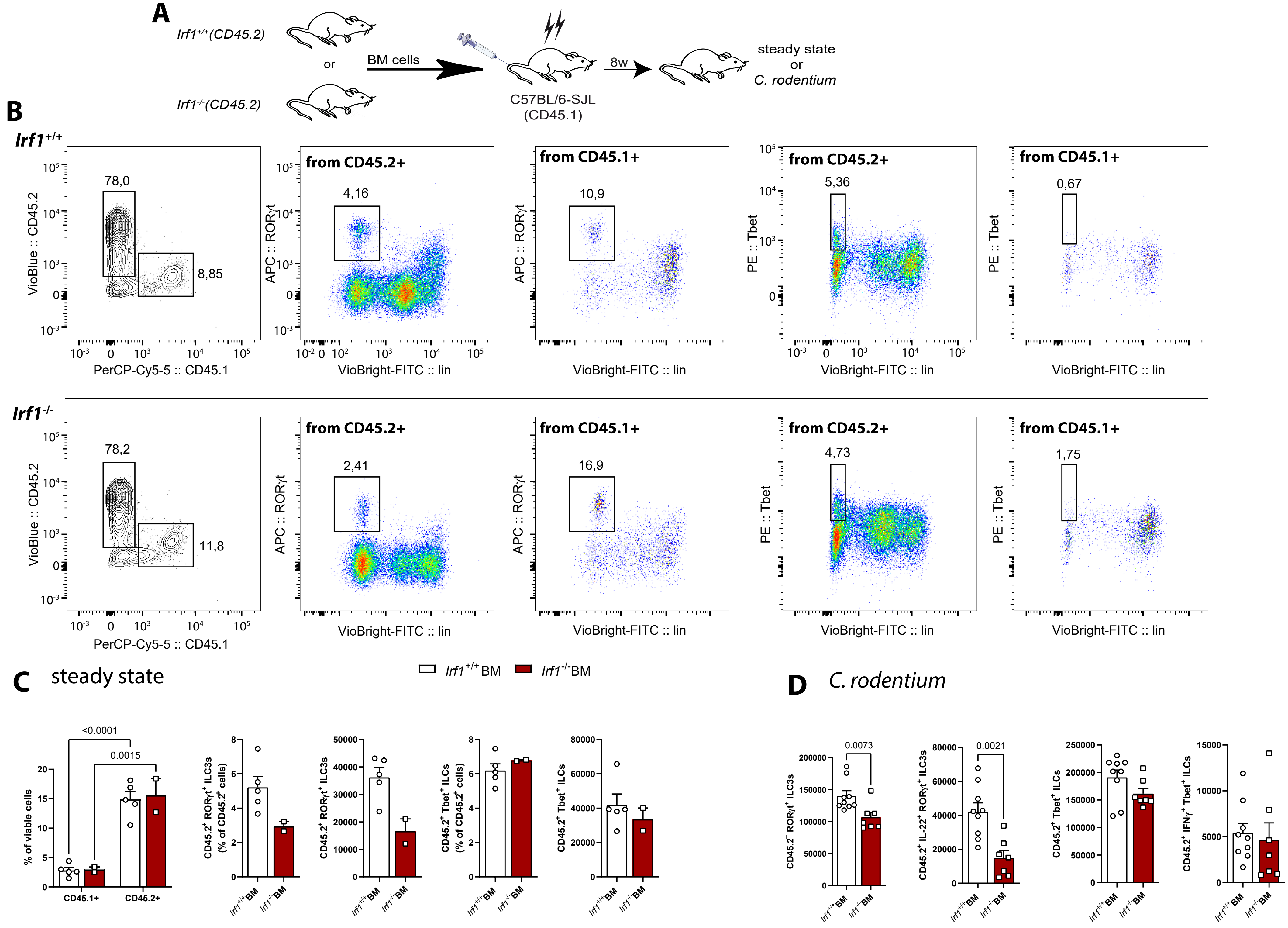

Supplementary Figure 2

Supplementary Figure 2

(A) Chimeric mice were generated by reconstitution of irradiated congenic C57BL/6-SJL mice ($CD45.1^{+}$) with bone marrow of *Irf1*$^{+/+}$ or *Irf1*$^{-/-}$ mice**. (B)** Gating strategy to characterize the reconstitution of RORγt^{+} and Tbet^{+} ILCs in LPMC by flow cytometry. **(C)** Analysis of ILC frequencies in the steady state (*Irf1*$^{+/+}$: n=5, *Irf1*$^{-/-}$: n=2) . **(D)**. Chimeras were infected with *C. rodentium* and at 9 dpi LPMCs were isolated and analyzed by flow cytometry (*Irf1*$^{+/+}$: n=9, *Irf1*$^{-/-}$: n=7). Data is expressed as mean ± SEM. Exact p values with ≤ 0.05 as defined by two-tailed ANOVA with Tukey's multiple comparison test (C) or Mann-Whitney U test (D) are provided in the figure plots. Source data are provided as a Source data file.

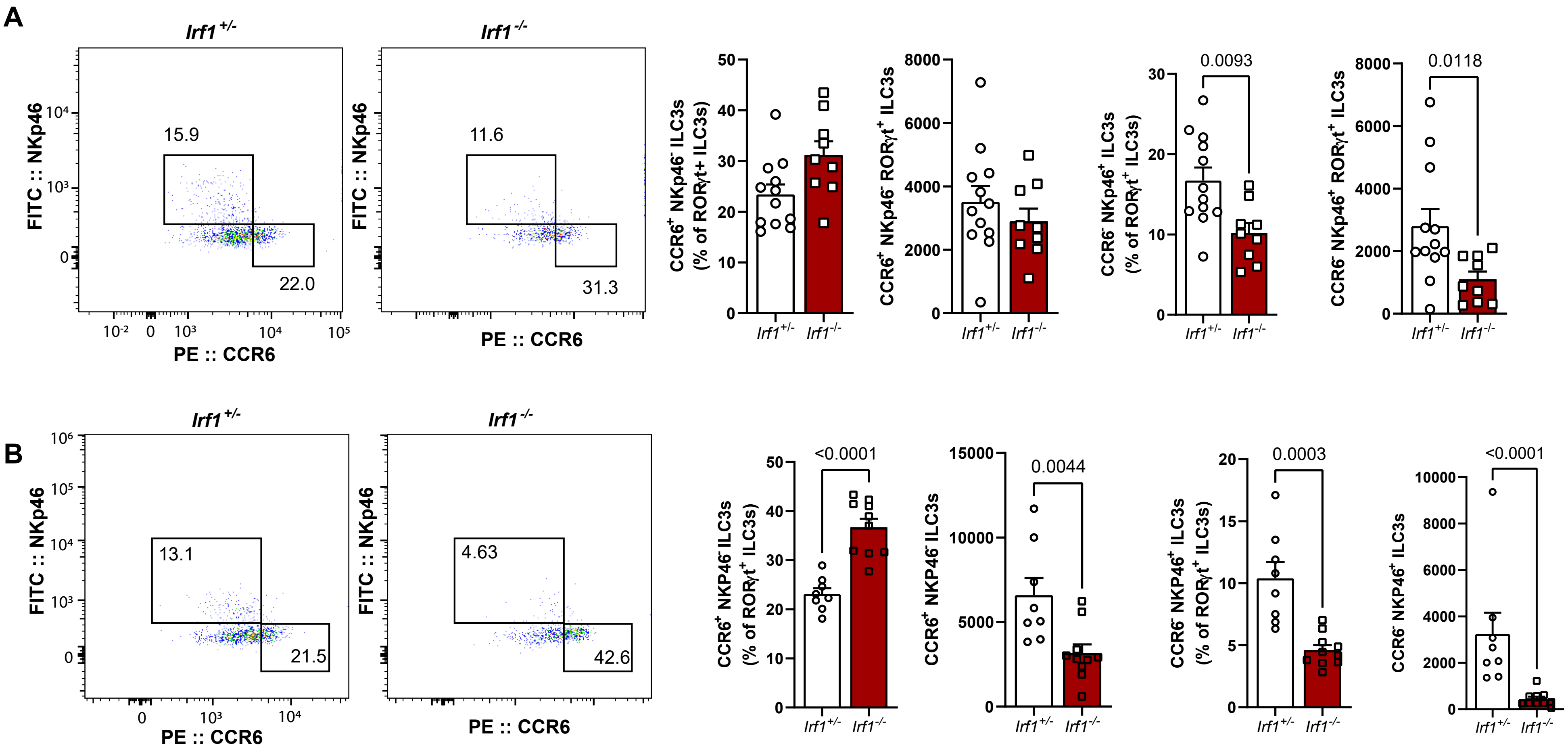

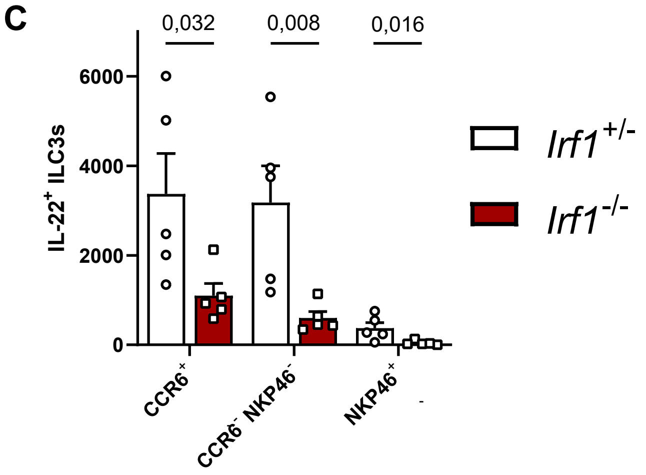

Supplementary Figure 3

Irf1$^{+/-}$ and *Irf1*$^{-/-}$ mice were left uninfected **(A)** or infected with *C. rodentium* for eight days **(B,C)**. Intestinal LPMCs were isolated and analyzed by flow cytometry. NKp46 and CCR6 were stained to determine the frequencies of CCR6^{+}NKp46^{-} and CCR6^{-}NKP46^{+} ILC3s (lin^{-}Thy1.2^{+}RORγt^{+} cells) or IL-22 producing ILC3 subtypes. (A) (*Irf1*$^{+/-}$: n=12, *Irf1*$^{-/-}$: n=9). (B) (*Irf1*$^{+/-}$: n=8, *Irf1*$^{-/-}$: n=10). (C) (*Irf1*$^{+/-}$: n=5/group. Data is expressed as mean ± SEM. Exact P values ≤ 0.05 as defined by two-tailed Mann-Whitney U test are provided in the figure plots. Source data are provided as a Source data file.

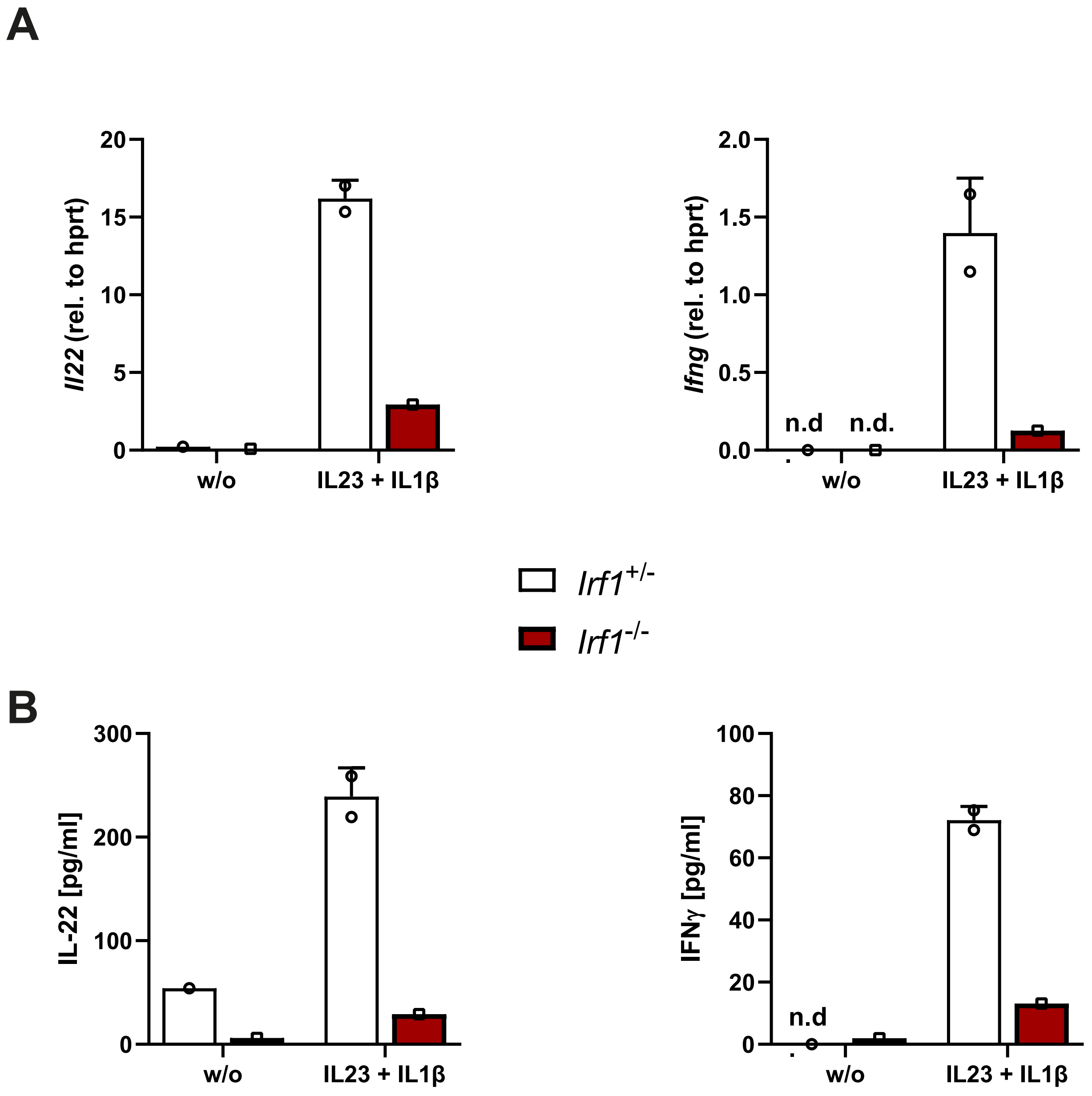

Supplementary Figure 4

Irf1$^{+/-}$ and *Irf1*$^{-/-}$ ILCs were sort-purified from LPMC before *in vitro* stimulation with IL-1β (20 ng/ml) and IL-23 (20 ng/ml) or medium. **(A)** Total RNA of ILCs was analyzed by specific qRT-PCR. **(B)** After 24 h, supernatants were collected to measure concentrations of IL-22 and IFN-γ by ELISA. Graphs represent data from three mice/group. Data is expressed as mean ± SEM. n.d. : not detectable. Source data are provided as a Source data file.

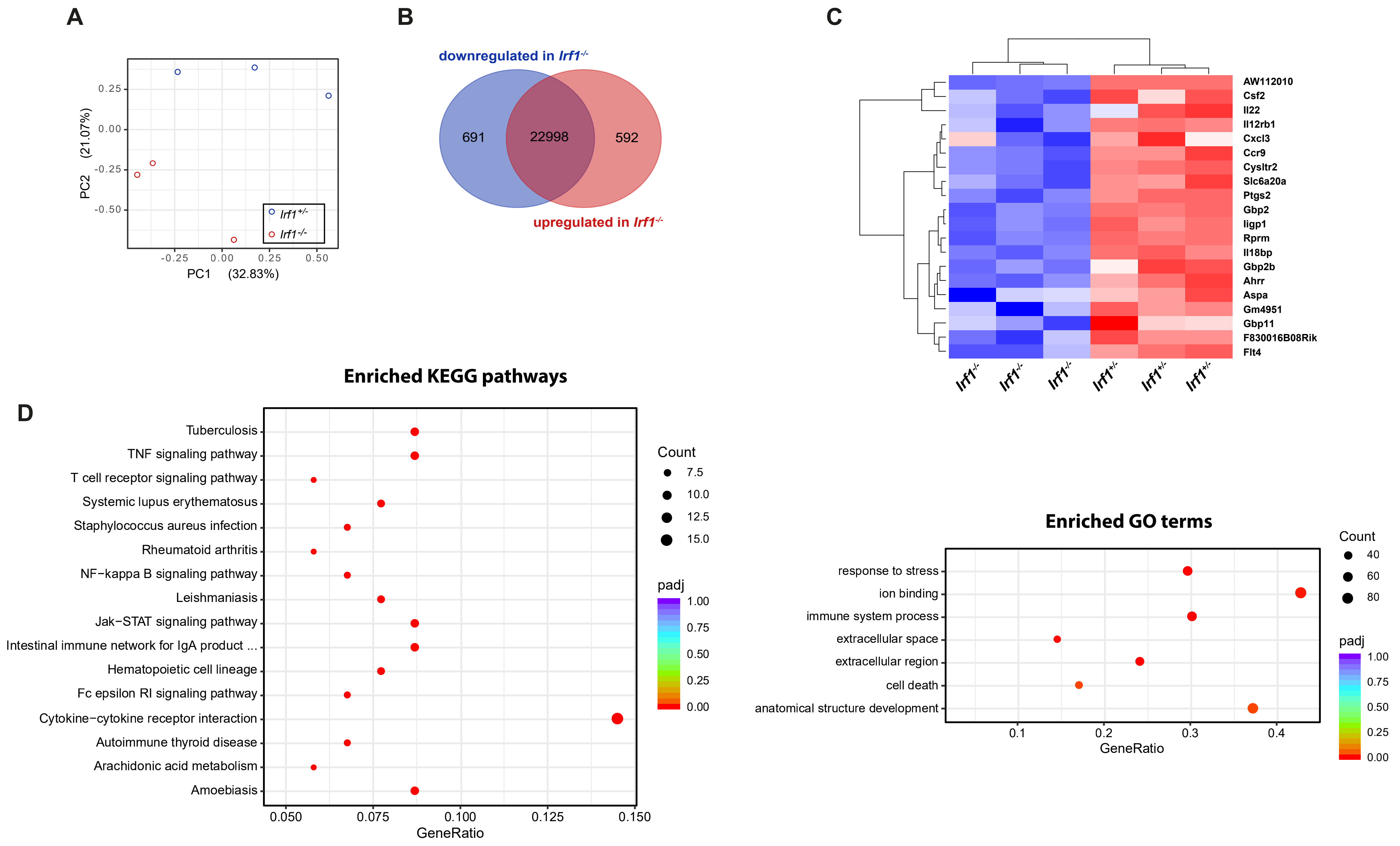

Supplementary Figure 5

(A-D) Analysis of gene expression profiling by bulk RNAseq of flow-sorted intestinal ILC1/ILC3s of *C. rodentium* infected *Irf1*$^{-/-}$ and *Irf1*$^{+/+}$ mice (9 dpi). Bulk RNAseq was conducted with three samples/group with cells sorted from two mice of the same genotype pooled to one sample. Fisher's exact test was used for identification of enriched pathways.

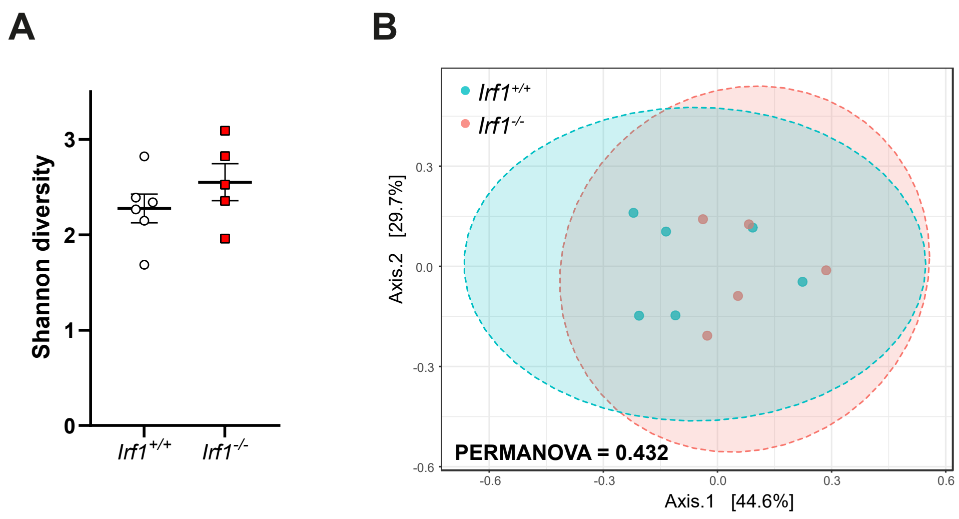

Supplementary Figure 6

(A,B) Fresh stool pellets of untreated, co-housed *Irf1*$^{+/+}$ (n=6) and *Irf1*$^{-/-}$ (n=5) mice were collected and prepared for 16S-based next generation sequencing. **(A)** Alpha-diversity is presented as Shannon diversity. **(B)** Principal coordinate analysis based on Bray-Curtis dissimilarity.

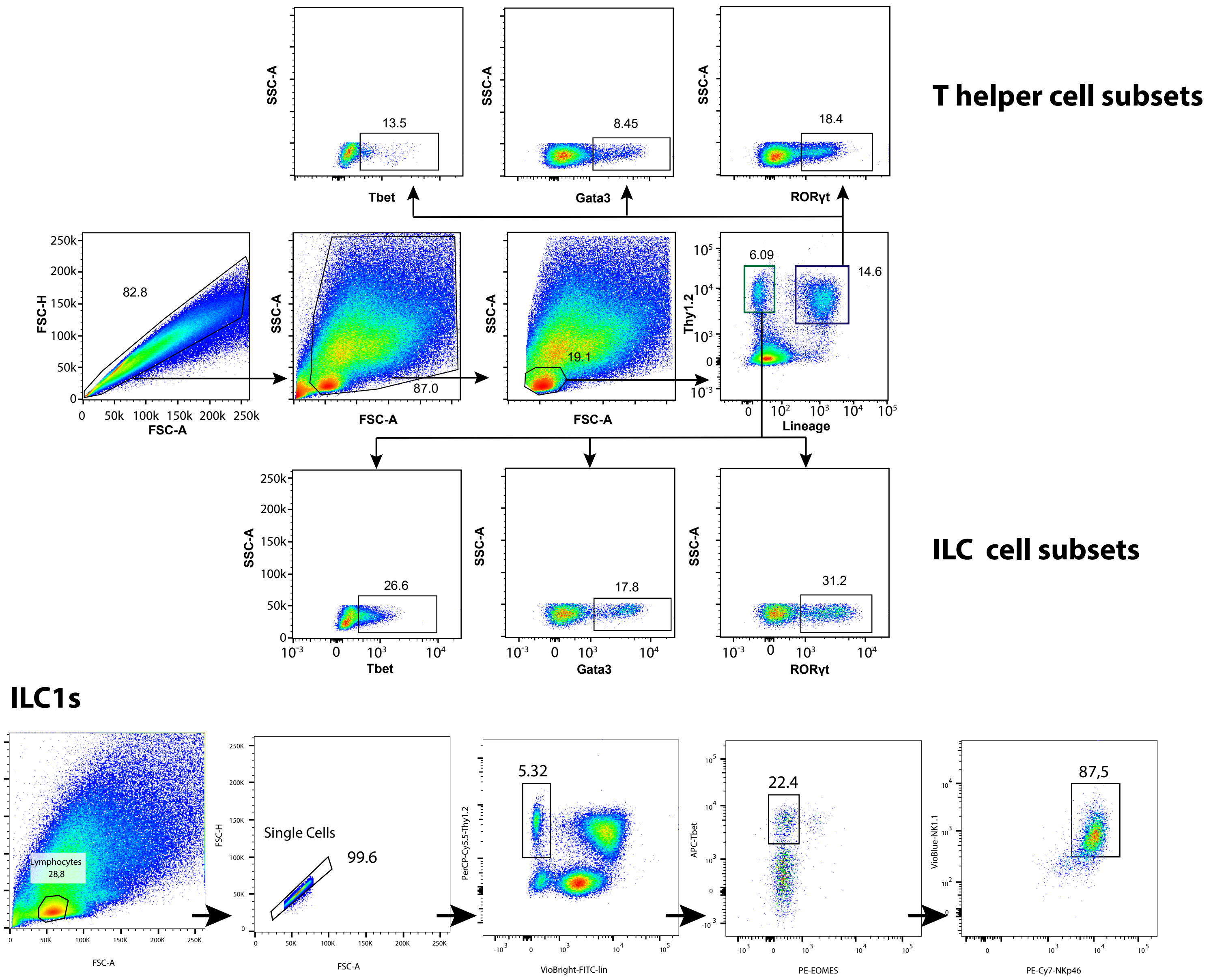

Supplementary Figure 7

Gating strategies to characterize the indicated lymphocyte population within intestinal LPMCs by multicolor flow cytometry.
