# Supplementary material for: Interferon regulatory factor 1 (IRF-1) promotes intestinal group 3 innate lymphoid responses during Citrobacter rodentium infection
Source: Nat Commun. 2022 Sep 29;13:5730. doi: 10.1038/s41467-022-33326-5 (PMC9522774; doi:10.1038/s41467-022-33326-5)
Supplement: Supplementary file 2 — Reporting Summary [file 41467_2022_33326_MOESM2_ESM.pdf]

nature portfolio

Corresponding author(s): Stefan Wirtz

Last updated by author(s): Jul 21, 2022

Reporting Summary

Nature Portfolio wishes to improve the reproducibility of the work that we publish. This form provides structure for consistency and transparency in reporting. For further information on Nature Portfolio policies, see our Editorial Policies and the Editorial Policy Checklist.

Statistics

For all statistical analyses, confirm that the following items are present in the figure legend, table legend, main text, or Methods section.

n/a	Confirmed	
[ ]	[x]	The exact sample size (*n*) for each experimental group/condition, given as a discrete number and unit of measurement
[x]	[ ]	A statement on whether measurements were taken from distinct samples or whether the same sample was measured repeatedly
[ ]	[x]	The statistical test(s) used AND whether they are one- or two-sided<br>*Only common tests should be described solely by name; describe more complex techniques in the Methods section.*
[x]	[ ]	A description of all covariates tested
[x]	[ ]	A description of any assumptions or corrections, such as tests of normality and adjustment for multiple comparisons
[ ]	[x]	A full description of the statistical parameters including central tendency (e.g. means) or other basic estimates (e.g. regression coefficient) AND variation (e.g. standard deviation) or associated estimates of uncertainty (e.g. confidence intervals)
[ ]	[x]	For null hypothesis testing, the test statistic (e.g. *F*, *t*, *r*) with confidence intervals, effect sizes, degrees of freedom and *P* value noted<br>*Give P values as exact values whenever suitable.*
[x]	[ ]	For Bayesian analysis, information on the choice of priors and Markov chain Monte Carlo settings
[x]	[ ]	For hierarchical and complex designs, identification of the appropriate level for tests and full reporting of outcomes
[x]	[ ]	Estimates of effect sizes (e.g. Cohen's *d*, Pearson's *r*), indicating how they were calculated

Our web collection on statistics for biologists contains articles on many of the points above.

Software and code

Policy information about availability of computer code

Data collection	Leica LAS AF version 2.7.3.9723, BD FacsDiva Software version 6.1.3
Data analysis	Graphpad prism version 8.3.0, Excel 2016, Flowjo 10.6, HISAT 2.05, FeatureCounts 1.6.4, Deseq2 1.22.2, GOseq 3.11, Fiji/ImageJ 1.5, R 4.2

For manuscripts utilizing custom algorithms or software that are central to the research but not yet described in published literature, software must be made available to editors and reviewers. We strongly encourage code deposition in a community repository (e.g. GitHub). See the Nature Portfolio guidelines for submitting code & software for further information.

Data

Policy information about availability of data

All manuscripts must include a data availability statement. This statement should provide the following information, where applicable:

- Accession codes, unique identifiers, or web links for publicly available datasets
- A description of any restrictions on data availability
- For clinical datasets or third party data, please ensure that the statement adheres to our policy

The RNA sequencing of this study have been deposited in the Sequence Read Archive (SRA) database of the NCBI under the Bioproject accession number: PRJNA705051 (https://www.ncbi.nlm.nih.gov/sra/PRJNA705051). All other data generated during the current study are available from the corresponding author on reasonable request. The source data underlying the generated figures are provided as a Source Data file.

Field-specific reporting

Please select the one below that is the best fit for your research. If you are not sure, read the appropriate sections before making your selection.

☒ Life sciences ☐ Behavioural & social sciences ☐ Ecological, evolutionary & environmental sciences

For a reference copy of the document with all sections, see nature.com/documents/nr-reporting-summary-flat.pdf

Life sciences study design

All studies must disclose on these points even when the disclosure is negative.

Sample size	Sample size estimation was carried out using prior data for expected sample means and standard deviations generated in murine Citrobacter rodentium infection models. Thereby, probability of a type 1 error was set at $p < 0.05$ and that of a type 2 error was set at $p< 0.2$. Sample sizes were similar in size to most existing studies in the same field.
Data exclusions	no data were excluded
Replication	Results of experiments were reliably reproduced at least 2 times and alternative experimental settings were used to test hypotheses.
Randomization	For administration experiments (IL-22versus mock), animals in each cage were randomly assigned to either vehicle or treatment groups. For in vitro and ex vivo experiments using primary cells allocation to treatment or control groups was done randomly.
Blinding	Blinding was used in multiphoton imaging and CFU determination on agar plates. Other analyses were non-blinded. Matching samples were collected and analysed under the same conditions.

Reporting for specific materials, systems and methods

We require information from authors about some types of materials, experimental systems and methods used in many studies. Here, indicate whether each material, system or method listed is relevant to your study. If you are not sure if a list item applies to your research, read the appropriate section before selecting a response.

Materials & experimental systems

n/a	Involved in the study	
☐	☒	Antibodies
☒	☐	Eukaryotic cell lines
☒	☐	Palaeontology and archaeology
☐	☒	Animals and other organisms
☒	☐	Human research participants
☒	☐	Clinical data
☒	☐	Dual use research of concern

Methods

n/a	Involved in the study	
☒	☐	ChIP-seq
☐	☒	Flow cytometry
☒	☐	MRI-based neuroimaging

Antibodies

Antibodies used

Anibodies and the dilutions used in experiments :
- anti-B220 Biotin, clone RA3-6B2, catalog # 13-0452-85, eBioscience, 1:50
- anti B220 FITC, clone REA755, catalog # 130-110-708, Miltenyi Biotec, 1:50
- anti CD11b APC-Vio770, clone REA592, catalog # 130-113-803, Miltenyi Biotec, 1:50
- anti CD11c APC-Vio770, clone REA754, catalog # 130-110-841, Miltenyi Biotec, 1:50
- anti CD16/CD32, clone 93, # 14-0161-86, eBioscience, 1:200
- anti CD127 PE, clone, A7R34, # 135010, BioLegend, 1:20
- anti CD196 (CCR6) PE, clone REA277,# 130-126-497, Miltenyi Biotec, 1:20
- anti CD212 (IL 12Rβ1) PE, clone 114, # 551974, BD Biosciences, 1:20
- anti CD3 Biotin, clone REA641, # 130-123-861, Miltenyi Biotec, 1:50
- anti CD326 (Ep-CAM) AF488, cloneG8.8, # 118210, BioLegend, 1:100
- anti CD45 VioBlue, clone REA737, # 130-110-802, Miltenyi Biotec, 1:50
- anti CD5 Biotin, clone REA421, # 130-106-200, Miltenyi Biotec, 1:20
- anti CD90.2 (Thy 1.2) PerCP-Vio700, clone 30-H12, # 130-102-204, Miltenyi Biotec, 1:20
- anti Citrobacter, clone ab37056, # ab37056, Abcam, 1:1000
- anti EOMES, clone REA116, # 130-102-419, Miltenyi, 1:10
- anti GR1 Biotin, clone RB6-8C5, # MA5-17969 , eBioscience, 1:333
- anti IFNγ APC, clone XMG1.2, # 17-7311-82, eBioscience, 1:158
- anti IL-17A PE-Cy7, clone TC11-18H10.1, # 506922, BioLegend, 1:20

• anti IL-22 PE, clone Poly5164, # 516404, BioLegend, 1:20
• anti IL-23R AF647, clone 753317, # FAB16861R, R&D systems, 1:66
• anti IRF-1, clone D5E4, # 8478, Cell Signaling, 1:50
• anti KLRG1 APC, clone 2F1, # 17-5893-82, eBioscience, 1:158
• anti NK1.1 BV421, clone PK136, # 108741, Biolegend, 1:20
• anti NKp46 FITC, clone REA815, # 130-112-357, Miltenyi Biotec, 1:50
• anti RORγt AF647, clone Q31-378, # 562682, BD Bioscience, 1:80
• anti SiglecF Biotin, clone REA798, # 130-112-171, Miltenyi Biotec, 1:50
• anti Tbet PE, clone eBio4B10, # 12-5825-82, eBioscience, 1:40
• anti Ter119 Biotin, clone Ter-119, # 130-120-828 Miltenyi Biotec, 1:50
• anti GATA3 PE, clone REA174, # 130-123-748 Miltenyi Biotec, 1:10

Validation

All antibodies are from commercial resources and have been selected based on prior publications the manufacturer's validation reports for the reported application.

• anti-B220 Biotin, clone RA3-6B2, catalog # 13-0452-85, eBioscience (https://www.thermofisher.com/order/genome-database/dataSheetPdf?producttype=antibody&productsubtype=antibody_primary&productId=13-0452-85&version=234)
• anti-B220 Biotin, clone RA3-6B2, catalog # 13-0452-85, eBioscience (https://www.thermofisher.com/order/genome-database/dataSheetPdf?producttype=antibody&productsubtype=antibody_primary&productId=13-0452-85&version=234)
• anti B220 FITC, clone REA755, catalog # 130-110-708, Miltenyi Biotec (https://www.miltenyibiotec.com/upload/assets/dataSheet_p40635_eng_GBR.pdf)
• anti CD11b APC-Vio770, clone REA592, catalog # 130-113-803, Miltenyi Biotec (https://www.miltenyibiotec.com/upload/assets/dataSheet_p41919_eng_GBR.pdf)
• anti CD11c APC-Vio770, clone REA754, catalog # 130-110-841, Miltenyi Biotec (https://www.miltenyibiotec.com/upload/assets/dataSheet_p40596_eng_GBR.pdf)
• anti CD16/CD32, clone 93, # 14-0161-86, eBioscience (https://www.thermofisher.com/order/genome-database/dataSheetPdf?producttype=antibody&productsubtype=antibody_primary&productId=14-0161-86&version=234)
• anti CD127 PE, clone, A7R34, # 135010, BioLegend (https://www.biolegend.com/en-us/products/pe-anti-mouse-cd127-il-7ralpha-antibody-6190?pdf=true&displayInline=true&leftRightMargin=15&topBottomMargin=15&filename=PE%20anti-mouse%20CD127%20(IL-7R%CE%B1)%20Antibody.pdf)
• anti CD196 (CCR6) PE, clone REA277,# 130-126-497, Miltenyi Biotec (https://www.miltenyibiotec.com/upload/assets/dataSheet_p69317_eng_GBR.pdf)
• anti CD212 (IL 12Rβ1) PE, clone 114, # 551974, BD Biosciences (https://www.bdbiosciences.com/content/bdb/paths/generate-tds-document.us.551974.pdf)
• anti CD3 Biotin, clone REA641, # 130-123-861, Miltenyi Biotec (https://www.miltenyibiotec.com/upload/assets/dataSheet_p66892_eng_GBR.pdf)
• anti CD326 (Ep-CAM) AF488, cloneG8.8, # 118210, BioLegend (https://www.biolegend.com/en-us/products/alexa-fluor-488-anti-mouse-cd326-ep-cam-antibody-4972?pdf=true&displayInline=true&leftRightMargin=15&topBottomMargin=15&filename=Alexa%20Fluor%C2%AE%20488%20anti-mouse%20CD326%20(Ep-CAM)%20Antibody.pdf)
• anti CD45 VioBlue, clone REA737, # 130-110-802, Miltenyi Biotec (https://www.miltenyibiotec.com/upload/assets/dataSheet_p40542_eng_GBR.pdf)
• anti CD5 Biotin, clone REA421, # 130-106-200, Miltenyi Biotec (https://www.miltenyibiotec.com/upload/assets/dataSheet_p33116_eng_GBR.pdf)
• anti CD90.2 (Thy 1.2) PerCP-Vio700, clone 30-H12, # 130-102-204 Miltenyi Biotec, (https://www.miltenyibiotec.com/upload/assets/dataSheet_p17756_eng_GBR.pdf)
• anti Citrobacter, clone ab37056, # ab37056, Abcam (https://www.abcam.com/ab37056.pdf?)
• anti EOMES PE, clone REA116, # 130-102-419, Miltenyi (https://www.miltenyibiotec.com/upload/assets/dataSheet_p24873_eng_GBR.pdf)
• anti GR1 Biotin, clone RB6-8C5, # MA5-17969 , eBioscience (https://www.thermofisher.com/order/genome-database/dataSheetPdf?producttype=antibody&productsubtype=antibody_primary&productId=MA5-17969&version=234)
• anti IFNγ APC, clone XMG1.2, # 17-7311-82, eBioscience (https://www.thermofisher.com/order/genome-database/dataSheetPdf?producttype=antibody&productsubtype=antibody_primary&productId=17-7311-82&version=234)
• anti IL-17A PE-Cy7, clone TC11-18H10.1, # 506922, BioLegend (https://www.biolegend.com/en-us/products/pe-cyanine7-anti-mouse-il-17a-antibody-6013?pdf=true&displayInline=true&leftRightMargin=15&topBottomMargin=15&filename=PE/Cyanine7%20anti-mouse%20IL-17A%20Antibody.pdf)
• anti IL-22 PE, clone Poly5164, # 516404, BioLegend (https://www.biolegend.com/en-us/products/pe-anti-mouse-il-22-antibody-6486?pdf=true&displayInline=true&leftRightMargin=15&topBottomMargin=15&filename=PE%20anti-mouse%20IL-22%20Antibody.pdf)
• anti IL-23R AF647, clone 753317, # FAB16861R, R&D systems (https://resources.rndsystems.com/pdfs/datasheets/fab16861r.pdf?v=20220721&_ga=2.258445130.1184628120.1658402489-2114996117.1599222515)
• anti IRF-1, clone D5E4, # 8478, Cell Signaling (https://www.cellsignal.de/datasheet.jsp?productId=8478&images=1&size=A4)
• anti KLRG1 APC, clone 2F1, # 17-5893-82, eBioscience (https://www.thermofisher.com/order/genome-database/dataSheetPdf?producttype=antibody&productsubtype=antibody_primary&productId=17-5893-82&version=234)
• anti NK1.1 BV421, clone PK136, # 108741, Biolegend (https://www.biolegend.com/en-us/products/brilliant-violet-421-anti-mouse-nk-1-1-antibody-7150?pdf=true&displayInline=true&leftRightMargin=15&topBottomMargin=15&filename=Brilliant%20Violet%20421%E2%84%A2%20anti-mouse%20NK-1.1%20Antibody.pdf)
• anti NKp46 FITC, clone REA815, # 130-112-357, Miltenyi Biotec (https://www.miltenyibiotec.com/upload/assets/dataSheet_p41455_eng_GBR.pdf)
• anti RORγt AF647, clone Q31-378, # 562682, BD Bioscience (https://www.bdbiosciences.com/content/bdb/paths/generate-tds-document.us.562682.pdf)
• anti SiglecF Biotin, clone REA798, # 130-112-171, Miltenyi Biotec (https://www.miltenyibiotec.com/upload/assets/dataSheet_p41127_eng_GBR.pdf)

• anti Tbet PE, clone eBio4B10, # 12-5825-82, eBioscience (https://www.thermofisher.com/order/genome-database/dataSheetPdf?producttype=antibody&productsubtype=antibody_primary&productId=12-5825-82&version=234)
• anti Ter119 Biotin, clone Ter-119, # 130-120-828 Miltenyi Biotec (https://www.miltenyibiotec.com/upload/assets/dataSheet_p71839_eng_GBR.pdf)
• anti GATA3 PE, clone REA174, # 130-123-748 Miltenyi Biotec (https://www.miltenyibiotec.com/upload/assets/dataSheet_p72084_eng_GBR.pdf)

Animals and other organisms

Policy information about studies involving animals; ARRIVE guidelines recommended for reporting animal research

Laboratory animals	The folowing mouse strains were used in the study :<br>C57BL/6 mice, C57BL/6 SJL mice, Rag2-/-Il2rg-/- mice, Irf1-/-, Irf1+/- mice (Matsuyama et al., 1993). Ifng-/-, Irf1fl/fl (Eucomm), Tie2-cre mice, Villin-cre mice. All mice were bred on a C57BL/6 background and kept in individually ventilated cages under a 12h light/dark cycle. Female and male mice at an age og 8-16 weeks were used in the study. Mice of different experimental groups were age- and sex-matched. Sterile drinking water and food were provided ad libitum. and littermates were used as controls.
Wild animals	no wild animals were used
Field-collected samples	not used
Ethics oversight	Regierung von Unterfranken, Würzburg, Germany

Note that full information on the approval of the study protocol must also be provided in the manuscript.

Flow Cytometry

Plots

Confirm that:

- [x] The axis labels state the marker and fluorochrome used (e.g. CD4-FITC).
- [x] The axis scales are clearly visible. Include numbers along axes only for bottom left plot of group (a 'group' is an analysis of identical markers).
- [x] All plots are contour plots with outliers or pseudocolor plots.
- [x] A numerical value for number of cells or percentage (with statistics) is provided.

Methodology

Sample preparation	Single cell suspensions from mesenteric lymph nodes were prepared through digestion with Collagenase B (0.25 mg/ml; Roche) and DNase I (0.05 mg/ml Roche) using a gentleMACS™ Octo Dissociator (program: 37c_m_SDK_1; Miltenyi Biotec) according to the manufacturer's recommendations. For the isolation of lamina propria mononuclear cells (LPMCs) colonic or ileal tissue was removed and cleaned from residual fat. Luminal contents were flushed out and the intestinal tissue was cut longitudinally and then laterally into pieces of 5 mm length. LPMCs were isolated with the lamina propria dissociation kit mouse from Miltenyi Biotec according to the manufacturer's instructions under use of a gentleMACS™ Octo Dissociator (Miltenyi Biotec) running the program m_intestine_01. After the isolation process, the cell suspension was proceeded to Percoll gradient centrifugation (40 %/80 %) for purification.
Instrument	LSRFortessa cell analyzer (BD Biosciences)
Software	Flowjo 10.6
Cell population abundance	0,5-2x10^5 cells/sample were recorded by the instrument.
Gating strategy	Cell sorting ILC1/ILC3s werde defined as CD45+ B220-, CD3-, CD5-, CD11b-, CD11c-, KLRG1-, CD127+,Thy1.2+ cells

- [x] Tick this box to confirm that a figure exemplifying the gating strategy is provided in the Supplementary Information.
